# FBXW7 and human tumors: mechanisms of drug resistance and potential therapeutic strategies

**DOI:** 10.3389/fphar.2023.1278056

**Published:** 2023-11-13

**Authors:** Wanqing Wang, Kaipeng Jiang, Xue Liu, Ju Li, Wenshuo Zhou, Chang Wang, Jiuwei Cui, Tingting Liang

**Affiliations:** Cancer Center, The First Hospital of Jilin University, Changchun, Jilin, China

**Keywords:** Fbxw7, human tumors, drug resistance, targeted therapy, ubiquitin-proteasome system (UPS)

## Abstract

Drug therapy, including chemotherapy, targeted therapy, immunotherapy, and endocrine therapy, stands as the foremost therapeutic approach for contemporary human malignancies. However, increasing drug resistance during antineoplastic therapy has become a substantial barrier to favorable outcomes in cancer patients. To enhance the effectiveness of different cancer therapies, an in-depth understanding of the unique mechanisms underlying tumor drug resistance and the subsequent surmounting of antitumor drug resistance is required. Recently, F-box and WD Repeat Domain-containing-7 (FBXW7), a recognized tumor suppressor, has been found to be highly associated with tumor therapy resistance. This review provides a comprehensive summary of the underlying mechanisms through which FBXW7 facilitates the development of drug resistance in cancer. Additionally, this review elucidates the role of FBXW7 in therapeutic resistance of various types of human tumors. The strategies and challenges implicated in overcoming tumor therapy resistance by targeting FBXW7 are also discussed.

## 1 Introduction

The incidence and mortality rates of malignant tumors are rapidly increasing worldwide (Bray et al., 2021). According to up‐to‐date data analysis that there will be approximately 1,958,310 new cancer cases and 609,820 deaths in 2023 in the United States (Siegel et al., 2023). Malignant tumors significantly contribute to human mortality and impose major financial burden on societal advancement. Current cancer therapies include surgical interventions, radiation therapy, and drug therapy (such as chemotherapy, hormone therapy, immunotherapy, and molecular targeted therapy), with drug therapy being the predominant approach for treating human cancers. Unfortunately, the emergence of drug resistance often leads to unfavorable prognoses for a majority of tumor patients. Statistical evidence underscores the direct or indirect implication of drug resistance in 80%–90% of deaths amongst tumor patients (Mansoori et al., 2017; Ramos et al., 2021). Therefore, it is crucial to elucidate the intricate mechanisms of tumor drug resistance and identify strategies for its circumvention.

Drug resistance in cancer is an extremely complex phenomenon and can be divided into intrinsic resistance and acquired resistance depending on the temporal stage of manifestation (Wang et al., 2019; Ramos et al., 2021). The main mechanisms of drug resistance in tumor cells encompass an array of processes, such as drug efflux, apoptosis inhibition, enhanced DNA repair, modifications of drug targets, epigenetic alterations, promotion of drug metabolism and detoxification, emergence of tumor heterogeneity, epithelial-mesenchymal transition (EMT), perturbations in tumor microenvironment (TME), the influence of cancer stem cells (CSCs) and autophagy (Mohammad et al., 2015; Li et al., 2016; Assaraf et al., 2019; Erin et al., 2020; Nussinov et al., 2021; Ramos et al., 2021; Qin et al., 2023). With the development of genome sequencing technology, researchers have found that the presence of driver or background mutations can, to a certain extent, predict resistance to anticancer therapies. Noteworthy genes and signaling pathways include AKT, Bcl-2, mTOR, MAPK, IGF, Notch, and NF-κB (Peled et al., 2013; Bailey et al., 2014; Sigismund et al., 2018; Krishna et al., 2019; Murugan, 2019; Lee et al., 2020; Gallyas et al., 2020; Kapoor et al., 2020; Sato et al., 2020). Recently, several studies have confirmed the important role of F-box and WD Repeat Domain-containing-7 (FBXW7) in the development of tumor resistance (Inuzuka et al., 2011; Wang et al., 2011; Wertz et al., 2011; Song et al., 2019; Chen et al., 2023a).

The ubiquitin-proteasome system (UPS) is the main pathway forprotein degradation in eukaryotic cells (Wang et al., 2013; Wang et al., 2014; Zhou Z. et al., 2015). Autophagy is primarily responsible for the degradation of most long-lived proteins and some cellular organelles (Lilienbaum, 2013). The short-lived, misfolded, and damaged proteins degradation is regulated by cascade of three component enzymes of the UPS including ubiquitin activating E1 enzyme, ubiquitin conjugating E2 enzyme and ubiquitin-protein E3 ligase, respectively (Tekcham et al., 2020; Yang et al., 2021). E3 ligase plays a central role in the protein ubiquitination process, where it determines the specificity of a substrate for degradation (Berndsen and Wolberger, 2014; Tekcham et al., 2020). FBXW7 (also known as FBW7 or hCDC4) is a component of the SKP1-CDc53/Cullin-F-box protein complex (SCF-type E3 ubiquitin ligase) (Akhoondi et al., 2007). As a well-established tumor suppressor, FBXW7 is the most frequently mutated member of the human F-box protein family (Akhoondi et al., 2007; Yeh et al., 2018). We summarize the general mechanism by which FBXW7 is involved in tumor resistance through the available literature and describe the development of FBXW7 resistance in a variety of human tumors. In addition, we discuss the potential clinical applications of targeting FBXW7 in the treatment of tumor resistance. Finally, we highlight the challenges faced in overcoming tumor resistance using FBXW7.

## 2 FBXW7 introduction

**FIGURE 1:**
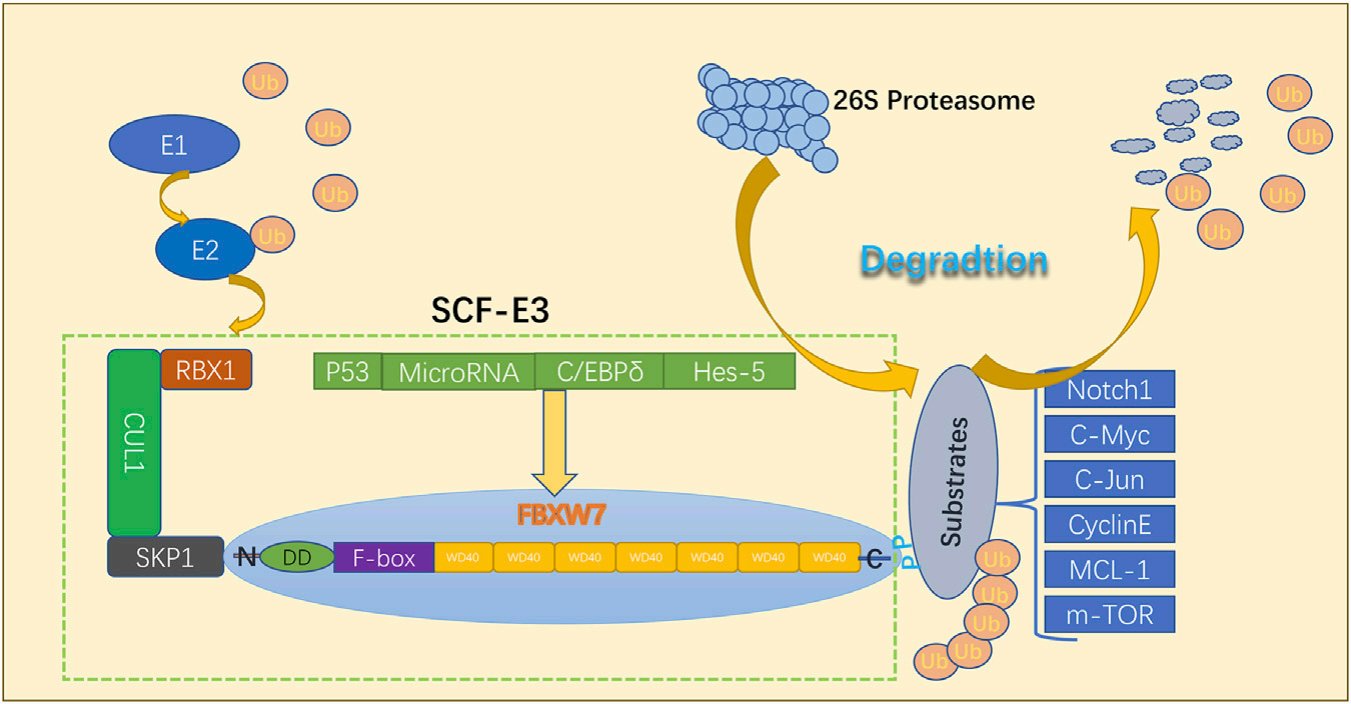
Structural characterization and functional mechanisms of FBXW7.

FBXW7, a member of the F-box protein family, is located on chromosome 4q31q3 (Zhao et al., 2012). The FBXW7 protein in eukaryotes can be divided into three isoforms: FBXW7α, FBXW7β, and FBXW7γ (Welcker et al., 2004; Davis et al., 2014). These isoforms have different N-terminal regions and cellular localization. FBXW7α, FBXW7β, and FBXW7γ are located in the nucleoplasm, cytoplasm, and nucleolus, respectively (Davis et al., 2014; Yeh et al., 2018). FBXW7α is thought to perform most FBXW7 functions (Yeh et al., 2018). The FBXW7α isoform may primarily control the cell cycle in proliferating cells, whereas FBXW7β-deficient cells are more sensitive to oxidative stress (Matsumoto et al., 2011; Cheng and Li, 2012; Cao et al., 2016) FBXW7γ has been reported to play key roles in regulating cell growth and c-Myc nucleolus abundance (Welcker et al., 2004). The common region of all isoforms contains three vital functional domains: (i) the dimerization domain that allows for isoform dimerization, (ii) the F-box domain that interacts with the SKP1-CUL1 complex, and (iii) the 7 tandem WD40 repeats that form a β-propeller structure and mediate substrate binding (Orlicky et al., 2003; Zhang and Koepp, 2006; Welcker et al., 2013; Davis et al., 2014; Lan and Sun, 2019). Subsequently, FBXW7 proteins capture phosphorylated substrates via the WD40 repeat domains, followed by their polyubiquitylation by the SCF E3 ligase (Nash et al., 2001; Sailo et al., 2019). After successive binding of multiple ubiquitin molecules to the substrate, degradation is instigated through the 26S proteasome as shown in Figure 1. It has been well-established that FBXW7 functions as a typical tumor suppressor by targeting a large number of critical human oncoproteins for ubiquitylation and proteasome degradation. These oncoprotein substrates include cyclin E, c-JUN, c-Myc, NOTCH-1, MCL-1 and KLF5, predominantly comprising transcription factors or key signaling molecules that regulate a wide range of cellular process involved in cell proliferation and tumor progression (Koepp et al., 2001; Strohmaier et al., 2001; O’Neil et al., 2007; Liu et al., 2010; Babaei-Jadidi et al., 2011; Inuzuka et al., 2011; King et al., 2013; Lan and Sun, 2019). FBXW7 is also regulated by a number of regulatory factors, including the tumor suppressor p53, C/EBP-δ (CCAAT/enhancer-binding protein-δ), Hes-5 (Hairy and Enhancer-of-split homologues 5), microRNAs (Yokobori et al., 2009; Balamurugan and Sterneck, 2013; Xia et al., 2017; Sailo et al., 2019). FBXW7 is inactivated by gene mutations, deletions, or promoter hypermethylation, which leads to oncoprotein accumulation (Akhoondi et al., 2010; Sailo et al., 2019; Fan et al., 2022). Numerous studies have indicated that FBXW7 often demonstrates abnormalities in various human cancers and may influence tumor biology as well as the development of drug resistance (Gong et al., 2018).

## 3 The role of FBXW7 in human tumors

FBXW7 has a high mutation rate in several human cancers. A meta-analysis of the COSMIC database revealed that the overall mutation rate of FBXW7 in all human malignancies was 7.79% (Forbes et al., 2017; Fan et al., 2022). Notably, FBXW7 is commonly rendered inactive by mutations, deletions, or promoter hypermethylation in some neoplasms, including hematologic malignancies, breast, colon, uterine, and lung cancer (Zhao et al., 2010; King et al., 2013; Yokobori et al., 2014; Kothari et al., 2016; Xiao et al., 2018; Cuevas et al., 2019). As mentioned previously, most known substrates regulated by FBXW7 are proto-oncoproteins. These proto-oncoproteins significantly affect the regulation of numerous crucial cellular processes, including cell proliferation, division, and differentiation. Therefore, aberrant FBXW7 expression is strongly associated with carcinogenesis, tumor progression, metastasis, poor outcomes in cancer patients, and resistance to treatment.

FBXW7 abnormalities manifest as pivotal drivers in the pathogenesis of tumorigenesis. The inactivation of FBXW7 leads to elevated levels of c-Myc and Cyclin E, resulting in the inability of cells to withdraw from the cell cycle, leading to uncontrolled proliferation (Fan et al., 2022). Tissue-specific ablation of FBXW7 has been shown to accelerate tumorigenesis in various mouse models. Wang et al. demonstrated that the inactivation of FBXW7 synergizes with activated AKT to induce intrahepatic cholangiocarcinoma (iCCA) in mice via c-Myc-dependent mechanisms (Wang J. et al., 2019). Another study showed that FBXW7 mutant mice exhibited thymic hyperplasia due to c-Myc accumulation and eventually developed thymic lymphoma (Onoyama et al., 2007). In human hematologic tumors, aberrant FBXW7 can act as an oncoprotein that promotes Notch1 signaling in adult T-cell leukemia (ATL) cells and may play an important role in the pathogenesis of ATL (Yeh et al., 2016; Yeh et al., 2018). Additionally, FBXW7 is a candidate cancer driver gene in chronic lymphocytic leukemia(CLL) (Close et al., 2019; Rossi, 2019). In a mouse intestinal cell model, the deletion of FBXW7 alone was insufficient to trigger intestinal malignancy. However, in the context of coexistence with other common mutations such as APC or P53 mutations, loss of FBXW7 accelerated intestinal tumorigenesis (Babaei-Jadidi et al., 2011).

FBXW7 significantly affects cancer aggressiveness and patient survival. A study determined that FBXW7 deficiency and certain FBXW7 mutations can promote the invasive and migratory capacity of esophageal squamous cell carcinoma (ESCC) cells via MAP4 overexpression and ERK phosphorylation (Pan et al., 2023). A recent meta-analysis showed that CRC patients with FBXW7 deletion had worse overall survival (Shang et al., 2021). Multifactorial COX regression analyses of several studies have also shown that FBXW7 deficiency is a prognostic marker in patients with CRC patients (Liu et al., 2018). Similar effects of FBXW7 on tumors were also observed in hepatocellular carcinoma, ovarian cancer, and T-ALL (Wang et al., 2015; Mihashi et al., 2017; Xu et al., 2020).

In addition, FBXW7 is strongly associated with treatment resistance in tumors (Wertz et al., 2011). In the subsequent sections, we will describe the mechanism of FBXW7 involvement in tumor drug resistance, its application in specific solid tumors, and therapeutic strategies targeting FBXW7.

## 4 General mechanism of FBXW7-mediated tumor drug resistance

### 4.1 FBXW7 inactivation promotes tumor cell escape from apoptosis

Many currently available anti-cancer therapies primarily involve the activation of cell death networks to eliminate malignant cells (Mohammad et al., 2015). However, in cancer, de-regulated apoptotic signaling allows cancer cells to escape this process leading to uncontrolled proliferation, thereby resulting in tumor survival, therapeutic resistance, and recurrence of cancer (Mohammad et al., 2015). Myeloid cell leukemia-1 (MCL-1) is a potent anti-apoptotic protein and a crucial member of the BCL-2 (B-cell CLL/Lymphoma 2) protein family. It has emerged as a critical survival factor in a broad range of human cancers (Mittal et al., 2021). Multiple E3 ubiquitin ligases are involved in the degradation of MCL-1 proteins, including FBXW7, MULE and β-TrCP (Zhong et al., 2005; Ding et al., 2007; Inuzuka et al., 2011; Senichkin et al., 2020). High-level expression of MCL-1 blocks apoptosis induced by various apoptotic stimuli and is associated with antitumor drug resistance (Quinn et al., 2011; Wang et al., 2021). Accumulation of the MCL-1 protein disrupts the homeostatic relationship between pro- and anti-apoptotic proteins, resulting in the inability to activate the cysteine asparaginase cascade that executes apoptosis (Wood, 2020; Sulkshane and Teni, 2022). Thus, abnormal FBXW7 expression may affect the efficacy of anticancer therapies through MCL-1 protein accumulation. It has been established that MCL-1 protein degradation is compromised observed subsequent to FBXW7 mutation or deletion within tumor cells, leading to resistance to anti-microtubule drugs such as paclitaxel and vincristine (Wertz et al., 2011). Recently, dysfunctional MCL protein degradation was ascertained in FBXW7-deficient CRC cells, thereby exhibiting resistance to the multi-kinase inhibitor (Tong et al., 2017a). Interestingly, restoration of FBXW7 expression or the use of a MCL-1 protein inhibitor can reverse the treatment resistance in CRC cells (Song et al., 2020). Thus, FBXW7 may be involved in tumor cell evasion of the apoptotic program and the consequent generation of therapy resistance by regulating the ubiquitination and hydrolysis of anti-apoptotic factors.

### 4.2 FBXW7 and epithelial–mesenchymal transition (EMT)

Epithelial–mesenchymal transition (EMT) is a malignant transformation process in which epithelial cells lose their properties and become mesenchymal cells (Lamouille et al., 2014; Don et al., 2019). Aberrant activation of EMT is associated with malignant properties of tumor cells during cancer progression and metastasis, including promoted migration and invasiveness, increased tumor stemness, and enhanced resistance to treatment (Huang et al., 2022). EMT activation can inhibit the sensitivity of tumor cells to antitumor drugs by altering the microenvironment, enhancing tumor cell anti-apoptosis, DNA repair, and altering drug metabolic pathways (Don et al., 2019; Song et al., 2020). FBXW7 expression regulates EMT in human cancers (Díaz and de Herreros, 2016). FBXW7 can inhibit the EMT process in part by downregulating EMT upstream transcription factors including such as c-Myc,Notch, mTOR, Snail 1, and zinc-finger E-box-binding homeobox 1 (ZEB1) and regulating the RhoA signaling pathway. Pertinently, the decrement of FBXW7 expression negates its inhibitory function (Cho et al., 2010; Fender et al., 2015; Yang et al., 2015; Li et al., 2016). Abnormalities in FBXW7 can contribute to the development of cancer drug resistance by indirectly affecting EMT. Research revealed that silencing the FBXW7 gene can promote the development of EMT and confer resistance to sorafenib and cisplatin in non-small cell lung cancer (NSCLC) (Xiao G. et al., 2018). *In vitro* studies revealed that elevated FBXW7 expression promotes the ubiquitin-mediated degradation of Snai1, which inhibits the EMT process and the renewal capacity of CSCs and consequently restores the sensitivity of NSCLC cells to the above drugs (Xiao G. et al., 2018). Similarly, Yu et al. (2014) found that FBXW7 expression levels are resistant to adriamycin in hepatocellular carcinoma (HCC) by affecting the EMT process. In FBXW7-expressing deficient colorectal cancer (CRC) cell lines, promotion of ZEB2-induced EMT mediates resistance to 5-fluorouracil (5-FU) and Oxaliplatin (OX) chemotherapeutics (Li et al., 2019).

### 4.3 FBXW7 and cancer stem cells (CSCs)

Cancer stem cells are a small subset of specialized tumor cells that can maintain tumor differentiation and self-renewal (Bayik and Lathia, 2021). Studies have revealed that the presence of cancer stem cells is an important reason why tumors exhibit treatment resistance (Zhou et al., 2017; Li et al., 2021).FBXW7 regulates CSCs self-renewal and cancer progression by reducing core transcription factor activity, activating specific signaling pathways, metabolic reprogramming, and the EMT program (Cremona et al., 2016; Zou et al., 2023). Aberrant FBXW7 expression leads to cancer stemness and poor clinical outcome. For example, FBXW7 is involved in the protection of CSCs from anticancer agent-induced cell death by regulating downstream transcription factors (e.g., ZEB2 and Snail 1) (Xiao et al., 2018; Li et al., 2019). However, this seems inseparable from the involvement of the EMT. Contrary to the aforementioned onco-suppressive role, FBXW7 targets positive regulators of the cell cycle for degradation, such as cyclin E and c-Myc, thereby maintaining CSCs in a quiescent and non-proliferative state (Essers and Trumpp, 2010; Shen et al., 2022). (PMID:20599449, 35515121) It is well known that current cytotoxic drugs mainly kill proliferating active cancer cells. These CSCs exiting the cell cycle adapt to their new microenvironment by acquiring mutations and epigenetic modifications that allow them to gain resistance to anticancer treatments (Tamamouna et al., 2022). (PMID: 36430404) (Fan et al., 2022) Therefore, based on the CSC-specific FBXW7-regulatory mechanism, low FBXW7 expression can expose CSCs to antitumor toxic drugs. Inhibition of FBXW7 expression in CSC after chemotherapy may be a promising strategy for eliminating colorectal CSC and improving their chemosensitivity to anticancer agents (Izumi et al., 2017). Similarly, a previous compelling study showed that abrogation of quiescence in leukemic initiating cells (LICs) through FBXW7 knockout increases their sensitivity to imatinib (Takeishi et al., 2013). These studies suggest that the regulation of CSCs quiescence through FBXW7 expression is an interesting strategy for enhancing the therapeutic sensitivity of certain tumors.

### 4.4 MicroRNA regulates FBXW7 expression level

FBXW7 contributes to the development of tumor resistance. Whether regulators (e.g., MicroRNA,P53, KLF5, Hes5) upstream of FBXW7 also indirectly regulate antitumor resistance remains unknown. MicroRNAs (MiRNAs) are a class of noncoding RNAs containing 19–24 nucleotides that play important roles in the regulation of cancer onset, progression, and anticancer drug resistance (Wu and Pfeffer, 2016). MiRNAs can regulate gene expression at the post-transcriptional level through translational repression and/or induction of mRNA degradation (Iorio and Croce, 2012). Various MiRNAs have been reported to target FBXW7 gene expression in human tumors, including MiR-25-3p, MiR-32, MiR-92b, MiR-96, MiR-155-3p, MiR-182, MiR-223 and MiR-367(58) (Fan et al., 2022). Therefore, MiRNAs may indirectly regulate anticancer drug sensitivity and resistance by affecting FBXW7 expression. Zhou et al. have identified a functional link between miR-223 and FBXW7 in gastric cancer. They found that the overexpression of miR-223 decreased the expression of FBXW7 and the sensitivity of GC cells to cisplatin, whereas inhibition of miR-223 restored the expression of FBXW7 and the sensitivity of GC cells to cisplatin (Zhou et al., 2015b). Similarly, the action of MiRNA on FBXW7 expression levels in the development of drug resistance has been demonstrated in other studies (Kumar et al., 2014; Eto et al., 2015; Hu et al., 2019; Wang et al., 2021; Feng et al., 2022). MiRNAs represent an important class of influential factors, even though this is not a direct mechanism. Thus, any of the above MicroRNAs are expected to act as regulators of FBXW7 expression and reduce the expression of FBXW7 substrates. Considering the wide range of roles in regulating FBXW7 in various cancers, MicroRNAs could serve as disease progression biomarkers and potential therapeutic strategies (Wu and Pfeffer, 2016; Lin et al., 2019) (PMID:26433073, 31546023).

### 4.5 Regulating the tumor microenvironment (TME) and immunotherapy

The tumor microenvironment (TME) is a complex ecosystem where cancer cells reside and is composed of fibroblasts, surrounding blood vessels, different immune cells, and the extracellular matrix (Liu et al., 2021). With tumor-infiltrating immune cells and cancer-associated fibroblasts (CAFs), the tumor microenvironment (TME) has emerged as a central player in cancer drug resistance, which constantly evolves during cancer progression and significantly affects treatment efficiency (Liu et al., 2022). FBXW7 is involved in immune evasion that occurs in anti-tumor immune responses, as well as in the regulation of the immune microenvironment, and its mutation or downregulation is more likely to lead to immunotherapy resistance (Xing et al., 2022). Mutation or loss of function of FBXW7 significantly reduces the infiltration of dendritic cells and immune cells, such as macrophages and CD8 T cells, in the tumor microenvironment, thereby promoting resistance to anti-PD-1 therapy (Gstalder et al., 2020; Ding et al., 2023). In addition to this, FBXW7 can regulate the tumor immune microenvironment by mediating the degradation of GSK-3β phosphorylated C/EBPδ, influencing macrophage polarization toward M2-type and modulating macrophage innate immune responses (Balamurugan et al., 2013; Zhong et al., 2020). M2-polarized macrophages participate in cancer initiation, development and metastasis by improving the invasive properties of tumor cells, immune suppression, hypoxia induction, angiogenic and lymphangiogenic regulation (Hughes et al., 2015; Boutilier and Elsawa, 2021).(PMID: 34209703,26269531) Yang’s study also demonstrated that the cancer cell progression induced by M2 macrophages was mechanistically linked to FBXW7-mediated MCL-1 stabilization in colon cancer cells (Lee et al., 2020) (PMID: 32444799). Thus FBXW7 may regulate the switching of macrophage phenotype in the tumor microenvironment. Shen et al. (2022) also found that FBXW7, by promoting eye absent homolog 2 (EYA2) degradation, could reduce the tumor mesenchymal phenotype and enables increased immune cell infiltration, thereby enhancing the response to anti-PD-1 therapy in a mouse tumor model. Mutations in FBXW7 have also been associated with sensitivity or resistance to immunotherapy in endometrial and pancreatic cancers, as analyzed using gene sequencing (Lin et al., 2021). Therefore, screening for FBXW7 status as a biomarker to predict tumor patient response to treatment with immune checkpoint inhibitors (ICBs) or as a target to improve the efficacy of immunotherapy deserves further exploration.

### 4.6 The role of FBXW7-mediated autophagy in tumor drug resistance

**FIGURE 2:**
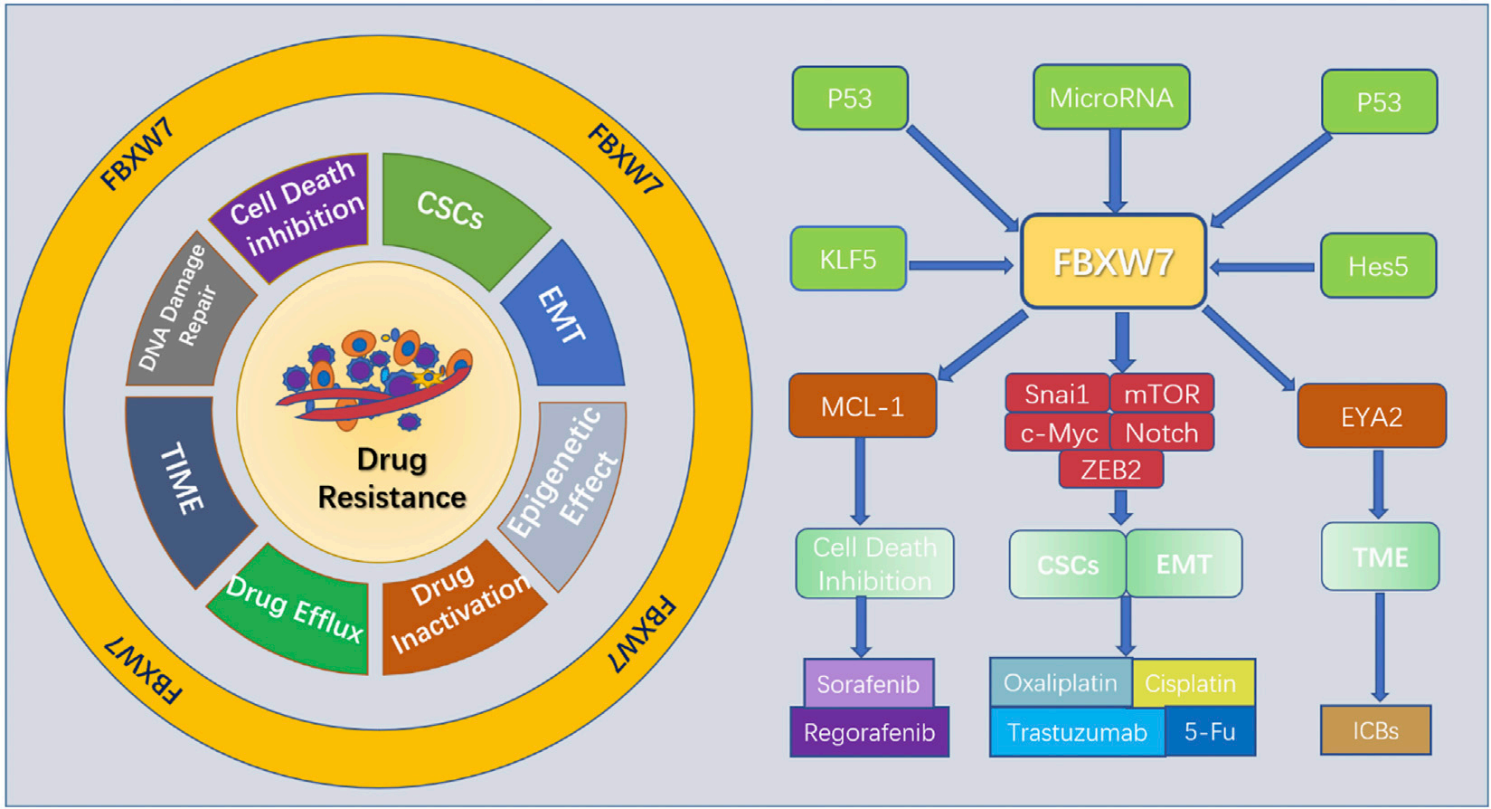
Mechanisms by which FBXW7 affects cancer drug resistance.

Autophagy is a lysosome-dependent pathway of self-degradation that plays a dual role in cancer, displaying both tumor suppressive and oncogenic activity (Kumar et al., 2015; Hu et al., 2023). Several studies have shown that the upregulated autophagy aids survival and enhances tumor resistance to anticancer therapy (Sui et al., 2013; Chang and Zou, 2020). Although the mechanisms by which autophagy promotes tumor drug resistance are not fully understood, the roles of FBXW7 in regulating autophagy to influence drug resistance are emerging. mTOR is well known as a key negtive regulator of autophagy, especially mTOR complex 1 (mTORC1) (Kim and Guan, 2015). However, the exact mechanism of how mTORC1 to regulate autophagy remains unclear. Considering this fact, Ye et al. proposed a new mode of the FBXW7-SHOC2-RPTOR axis in control of MTORC1 activity that affects autophagy (Xie and Sun, 2019). SHOC2 was found to be a substrate of FBXW7 and subject to FBXW7-mediated ubiquitination and degradation (Xie et al., 2019). It was also found that SHOC2 competes with mTOR to bind Raptor in a dose-dependent manner (Xie et al., 2019; Xie and Sun, 2019). Apparently, autophagy is under the regulation of FBXW7 that targets SHOC2. Another downstream target of FBXW7, MCL-1, is also thought to have a role in regulating autophagy. In patients with oral squamous cell carcinoma (OSCC) with reduced mRNA and protein levels of FBXW7, Sun’s found that the levels of MCL-1 expression increased and the mRNA encoding autophagy-associated proteins, including Beclin1, autophagy related 7, and microtubule-associated protein light chain 3 declined (Sun et al., 2022). This indicates that FBXW7 influences autophagy through MCL-1 in OSCC. Consistent with the above finding, Qiu’s study also reported that enhanced FBXW7 expression promotes autophagy in both OSCC cells and xenograft tumor model (Qiu et al., 2023). Furthermore, the interaction between MCL-1 and autophagy can produce different outcomes on apoptosis or cell survival. The investigators found that cell death-associated BECN1-dependent autophagy was inhibited in fludarabine -resistant (FdR) cells with sustained MCL-1 levels (Sharma et al., 2013). By analyzing *in vitro* experiments, found that autophagy regulated Erastin -induced ferroptosis in ALL cells via the FBXW7-VDAC3 axis Zhu et al. (2021). In addition, certain MicroRNAs have been reported to enhance cancer drug resistance via inducing FBXW7-mediated autophagy (Wang et al., 2020; Feng et al., 2022). These results suggest a clear relationship between FBXW7 and autophagy in the development of tumor drug resistance. Inhibiting autophagy by targeting FBXW7 may provide promising strategies to overcome drug resistance. The mechanisms by which FBXW7 affects cancer resistance are shown in Figure 2.

## 5 FBXW7 is involved in treatment resistance in multiple tumors

### 5.1 Non-small cell lung cancer (NSCLC)

Analysis of the Cancer Genome Atlas data revealed that deletion of FBXW7 occurred in 30.9% of lung adenocarcinomas and 63.5% of lung squamous carcinomas (Xiao et al., 2018). NSCLC patients with low FBXW7 expression not only have more aggressive tumors but also exhibit worse treatment efficacy and clinical prognosis (Yokobori et al., 2014). Reduced FBXW7 expression promotes NSCLC resistance to gefitinib, which could be reversed by combination therapy with gefitinib and rapamycin (an mTOR inhibitor) (Xiao et al., 2018). Furthermore, defective FBXW7 expression in NSCLC was found to be associated with paclitaxel resistance (Yokobori et al., 2014). MS-275, a histone deacetylase inhibitor, can reverse paclitaxel resistance in NSCLC cells with low or absent FBXW7 expression (Yokobori et al., 2014). As mentioned previously, FBXW7 overexpression can significantly enhance the chemosensitivity of NSCLC cells to cisplatin by interfering with EMT (Yu et al., 2013). Additionally, miR-223 was found to mediate autophagy by targeting FBXW7, which can also lead to cisplatin resistance in NSCLC (Wang et al., 2020). Anti-EGFR monoclonal antibody (such as gefitinib and erlotinib)-resistant NSCLC specimens show downregulation of FBXW7, which is related to the reduced degradation of MCL-1. Activation of FBXW7 promotes MCL-1 degradation and restores the sensitivity of drug-resistant NSCLC cells to targeted therapy (Ye et al., 2017). Recent studies have suggested that PD-1/PD-L1 protein abundance and stability may be regulated by ubiquitin-mediated proteasomal degradation (Ding et al., 2023). FBXW7 status can serve as a biomarker to predict patient response to anti-PD-1 immunotherapy in NSCLC, and an elevated expression of FBXW7 increases sensitivity to anti-PD-1 immunotherapy (Liu et al., 2022).

### 5.2 Colorectal cancer (CRC)

The mutation rate of FBXW7 in colorectal cancer is the second highest among all human malignancies (7.73%) (Yeh et al., 2018). Similarly, aberrant FBXW7 expression significantly affects CRC treatment resistance. Mutations in FBXW7 reduce phosphorylated P53 degradation, which is involved in oxaliplatin resistance in CRC cells (Li et al., 2015). It has also been found that cryptochrome 2 (CRY2) is negatively regulated by FBXW7 and is overexpressed in samples from chemotherapy-resistant CRC patients (Fang et al., 2015). High FBXW7 expression downregulates CRY2 and increases colorectal cancer cell sensitivity to oxaliplatin (Fang et al., 2015). An *in vitro* study also found that FBXW7-deficient CRC cells are more tolerant to the DNA-damaging agent 5 fluorouracil (5-FU) (Lorenzi et al., 2016). In addition, FBXW7 mutations were unable to mediate the degradation of MCL-1, which led to the resistance of CRC cells to targeted drugs (regorafenib, trametinib) (Tong et al., 2017a; Lin et al., 2020). In contrast, restoration of FBXW7 expression promotes MCL-1 degradation and reverses therapeutic resistance in CRC cells (Song et al., 2020). Gene sequencing has revealed that mutations in FBXW7 are significantly associated with resistance to anti-EGFR therapy (cetuximab or panitumumab) in CRC (Lupini et al., 2015). However, the exact underlying mechanism requires further investigation. A recent study using MC38, a colon carcinoma cell line syngeneic to C57BL/6 mice that is partially sensitive to anti-PD-1 treatment, found that the deletion of FBXW7 significantly reduced the response of MC38 to anti-PD-1 therapy (Gstalder et al., 2020). However, only one *in vitro* study strongly predicted the importance of FBXW7 in colorectal cancer immunotherapy. It is conceivable that the FBXW7 gene has a broad impact on CRC treatment resistance, from chemotherapy to targeted therapy and immunotherapy.

### 5.3 Hematological malignancies

Accumulating evidence indicates that the aberrant expression of FBXW7 is associated with drug resistance of hematological malignancies. FBXW7 mutation is found in 8%–12% of patients with T-ALL (T-lymphoblastic acute leukemia) and have been associated with a therapeutic response to the use of glucocorticoids (Kraszewska et al., 2013). A study showed that ubiquitin degradation of glucocorticoid receptor alpha (GRα) is regulated by FBXW7 (Malyukova et al., 2013). In primary T-ALL, loss of FBXW7 function leads to upregulation of GRα, which enhances glucocorticoid sensitivity (Wilkinson et al., 2018). Aberrant NOTCH1 activation signaling pathway is a major oncogenic driver of T-ALL (Toribio and González-García, 2023). Recognizing this, researchers have found inhibition of NOTCH signaling in T-ALL with Gamma-Secretase inhibitors (GSIs) to be a particularly attractive targeted therapeutic strategy (Hales et al., 2014). Although novel GSI agents have recently entered clinical trials, their therapeutic efficacy in T-ALL patients has not been established due to the presence of drug resistance. According to Jennifer et al., FBXW7 mutations were found to maintain NOTCH1 signaling in T-ALL and confer resistance to GSI (O’Neil et al., 2007). Notably, MiR-223 inhibition can increase FBXW7 levels in T-ALL cell lines and prevent resistance to GSI (Kumar et al., 2014). This suggests that MiR-223 may have therapeutic promise in targeted therapeutic regimens. In adult T cell leukemia (ATL), Yeh et al. (2020) found that mutations in FBXW7 may confer resistance to BET inhibitors in tumor cells. (PMID: 32907612) Resistance occurs due to increased phosphorylation and activation of c-Myc as a result of the inability of the FBXW7 mutation to target BRAF for degradation (Yeh et al., 2020). Recent evidence also suggests that FBXW7 protein accumulation contributes to the cytotoxic effects of BET inhibitors in T-ALL cell lines (Jiménez-Izquierdo et al., 2023). FBXW7 maintains quiescence in leukemia stem cells (LSCs) of chronic myeloid leukemia (CML) (Takeishi and Nakayama, 2016). In a mouse model of CML, it has been found that FBXW7-deficient leukemia-initiating cells (LICs) are more sensitive to imatinib (Takeishi et al., 2013; Eid et al., 2023). Inhibition of FBXW7 appears to be a potential option to improve treatment response in CML.

### 5.4 Gastric cancer (GC)

Studies have shown that patients with GC and FBXW7 inactivation have more aggressive tumors and worse prognosis (Yokobori et al., 2009; Hou and Deng, 2015). As previously mentioned, Zhou et al. discovered a functional link between miR-223 and FBXW7 in GC, suggesting that miR-223 promotes the development of cisplatin resistance in GC cells by targeting FBXW7 (D et al., 2018). In addition, miR-223 overexpression also reduced the levels of FBXW7 in GC, leading to proliferation, invasion, and in vitro-induced trastuzumab chemoresistance in GC cells (Eto et al., 2015).

### 5.5 Other tumors

The mutation rate of FBXW7 in patients with melanoma is 8% (Aydin et al., 2014). Similar to CRC, preclinical studies and case reports have shown that deletion of FBXW7 is associated with resistance to anti-PD-1 immunotherapy in melanoma patients (Iraz et al., 2017; Gstalder et al., 2020). In addition, FBXW7 is strongly associated with tumor immune cell infiltration and immunotherapeutic responses in renal cell carcinoma(RCC) (Xing et al., 2022). NFAT1 is a member of the nuclear factor of activated T cell (NFAT) family, which is involved in many aspects of cancer, including carcinogenesis, cancer metastasis, formation of the tumor microenvironment, and immunotherapeutic response (Jiang et al., 2019). In RCC patients, the protein levels of FBXW7 were negatively correlated with those of NFAT1, which might be the substrate of FBXW7 in RCC cells (Liu et al., 2022). Thus, it has the potential to enhance the anti-PD-1 immunotherapeutic effects and improve sunitinib resistance by targeting the FBXW7–NFAT1 axis in RCC (Xing et al., 2022; Liu et al., 2022c; Chen et al., 2023a). In hepatocellular carcinoma (HCC), miR-25 increases HCC resistance to sorafenib by regulating FBXW7-induced autophagy (Feng et al., 2022). Furthermore, in pancreatic cancer (PC), FBXW7 silencing significantly enhances the accumulation of MCL1 in PC cells and resistance to gemcitabine and paclitaxel (Ishii et al., 2017). FBXW7 overexpression enhances glioblastoma cell sensitivity to temozolomide (TMZ) by downregulating Aurora B, MCL-1, and Notch-1 (Lin et al., 2018). In addition, in nasopharyngeal carcinoma (NPC), FBXW7-deficient NPC cells were found to express multidrug-resistant proteins (MRPs) and exhibit cisplatin resistance (Song et al., 2015). In contrast, increased FBXW7 expression was observed in the cisplatin-sensitized NPC cells. The role of FBXW7 in human tumor resistance are summarized in Table 1.

**TABLE 1:** FBXW7 is involved in the development of drug resistance in various human tumors.

Cancer types	Drug	Mechanism	Reference
NSCLC	Gefitinib	FBXW7- mTOR	Xiao et al. (2018b)
	MS-275, Taxol	FBXW7-TOP2A(Topoisomerase)/MCL1	Yokobori et al. (2014)
	Cisplatin	FBXW7-EMT	Yu et al. (2013)
	Cisplatin	MiR-223-FBXW7-Autophagy	Wang et al. (2020)
	Gefitinib	FBXW7-MCL-1	Ye et al. (2017)
	ICBs	FBXW7-PD-1	Liu et al. (2022b)
CRC	Oxaliplatin	phospho-p53(Ser15)/FBXW7	Li et al. (2015)
	Oxaliplatin	FBXW7-CRY2	Fang et al. (2015)
	5-FU	Unclear	Lorenzi et al. (2016)
	Regorafenib, Hsp90 Inhibitors, Trametinib	FBXW7-MCL-1	Tong et al. (2017b), Tong et al. (2017a), Lin et al. (2020)
	Cetuximab Panitumumab	Unclear	Lupini et al. (2015)
	ICBs	FBXW7/ dsRNA sensing	Gstalder et al. (2020)
GC	Cisplatin, Trastuzumab	MiR-223-FBXW7	Eto et al. (2015), Zhou et al. (2015a)
T-ALL	Glucocorticoid	FBXW7- GRα	Malyukova et al. (2013), Wilkinson et al. (2018)
	GSIs	FBXW7- Notch 1	Hales et al. (2014)
ATL	BET inhibitors	FBXW7-BRAF	Yeh et al. (2020)
CML	imatinib	FBXW7-LSCs	Takeishi et al. (2013), Eid et al. (2023)
Melanoma	Pembrolizumab	Tumor immune microenvironment	Gstalder et al. (2020)
(RCC) Renal cell cancer	ICIs, Sunitinib	FBXW7- NFAT1-PD-L1	Liu et al. (2022c)
HCC	Sorafenib	MiR-25-FBXW7	Feng et al. (2022)
PC pancreatic cancer)	Gemcitabine, Paclitaxel	FBXW7-MCL-1	Ishii et al. (2017)
Glioblastoma	TMZ (temozolomide)	FBXW7-(Aurora B/MCL-1/Notch-1)	Lin et al. (2018)
NPC	Cisplatin	FBXW7-MRP	Song et al. (2015)

## 6 Potential therapeutic strategies targeting FBXW7

**FIGURE 3:**
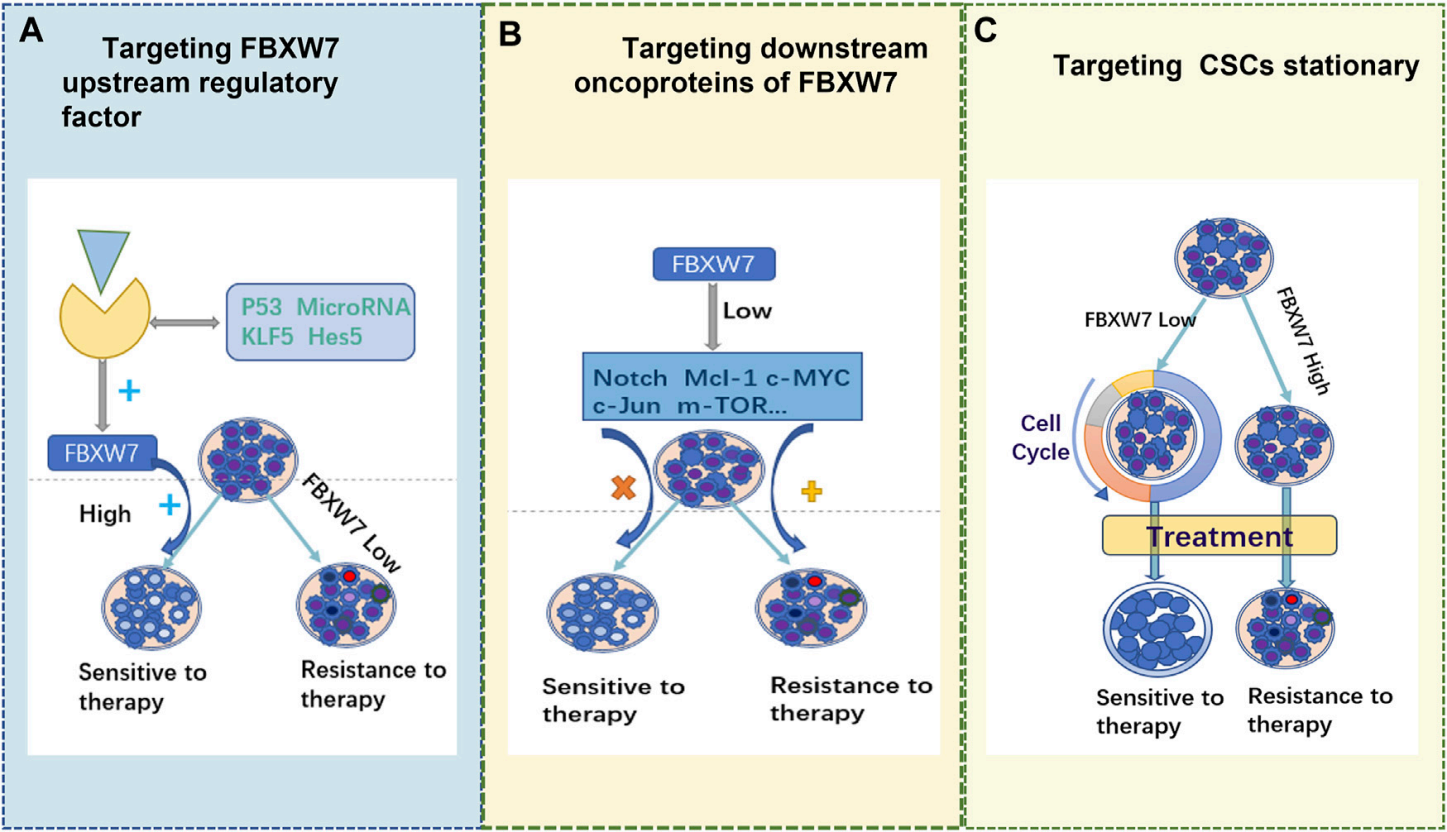
Three potential therapeutic strategies.

We have summarized some of the mechanisms by which FBXW7 contributes to treatment resistance and its application in various types of solid tumors. The deletion or aberrant FBXW7 expression results in a low rate downstream oncoprotein degradation. This leads to the accumulation of specific oncoproteins, thereby triggering drug resistance. Therefore, altering the expression level of FBXW7 may be a promising approach for reversing drug resistance. However, to date, no suitable drugs against FBXW7 have been developed. We propose the following promising strategies as shown in Figure 3. 1) targeting upstream regulators to promote FBXW7 reactivation, 2) targeting FBXW7 downstream pro-oncogenic proteins, and 3) inhibiting FBXW7 resistance in certain cancers. In addition, the limitations and challenges of targeting FBXW7 for antitumor drug resistance are discussed.

### 6.1 Targeting FBXW7 upstream regulators

The expression of FBXW7 is regulated by various factors, including P53, Pin1, C/EBP-δ, Hes-5, Numb4, and microRNA (Lixia et al., 2014). Therefore, restoring FBXW7 expression by designing drugs that target its upstream regulators may be a promising approach. P53 is one of the key regulators of FBXW7, and targeting the P53 signaling pathway can indirectly regulate the expression level of FBXW7 (Mao et al., 2004; Sailo et al., 2019). The main mechanism of P53 inactivation in human cancers is closely related to regulatory proteins such as MDM2 or MDM4 (also known as MDMX) (Cheok et al., 2011; Duffy et al., 2022). Thus, it is possible to maintain FBXW7 expression by designing P53 interaction blockers with MDM2 or MDM4 to prevent the degradation of wild type P53. In fact, several low-molecular-weight compounds that have been shown to block the binding between p53 and MDM2 (e.g., Nutlin-3a) have also been shown to improve the response to anticancer drugs in cancer cells *in vitro* (Cheok et al., 2011; Guo et al., 2017; Essmann et al., 2012). However, these experimental drugs are not used in clinical practice due to their strong side effects and limited efficacy. Now, more potent and safer P53-MDM2 blockers are currently being tested in clinical trials (Gupta et al., 2019). Although much evidence have shown that activated P53 can directly bind and activate FBXW7 gene expression, there may be a more complex (Mao et al., 2004; Yokobori et al., 2009). (PMID:15592418, 19366810) In some cases, FBXW7 can regulate the stability of P53 (Tripathi et al., 2019). (PMID:31346036) It was found that after DNA damage, FBXW7 can mediate P53 degradation, which allows cells to resume proliferation and may have a potentially detrimental effect on cancer outcomes (Elizabeth Caldon, 2020; Galindo-Moreno et al., 2020). (PMID:32316282, 33070871) It has also been suggested that the use of FBXW7 inhibitors together with DNA-damaging drugs may increase P53 levels and thus inhibit cell proliferation. However, this treatment requires further validation of its therapeutic efficacy and feasibility.

Another way to restore FBXW7 expression involves targeting the FBXW7-microRNA axis in tumor cells. miR-223 is overexpressed in cancer cells and participates in the development of resistance to multiple anticancer drugs by down-regulating FBXW7 expression (Wang et al., 2020; Ding et al., 2018; Zhou et al., 2015b; Eto et al., 2015; Zhang et al., 2017). Genistein was found to affect the biological behavior of pancreatic cancer cells by increasing FBXW7 expression through the downregulation of miR-223 (Ma et al., 2013). This pathway may also regulate drug resistance in pancreatic cancer cells. Another example is that downregulation of miR-188-5p by honokiol enhances doxorubicin sensitivity through FBXW7/c-Myc signaling pathway in human breast cancer (Yi et al., 2021). Therefore, enhancing the active expression of FBXW7 by targeting upstream regulators could be a novel approach for cancer drug resistance therapy.

### 6.2 Targeting key oncoproteins downstream of FBXW7

FBXW7 mediates the ubiquitination and hydrolysis of numerous oncoproteins, including key factors involved in cancer drug resistance. Deletion of FBXW7 leads to the accumulation of the pro-survival factor MCL-1, which confers chemoresistance to cancer cells. Thus, inhibition of MCL-1 expression or function may restore drug sensitivity in cancer cells. In CRC, MCL-1 inhibitors can be used to overcome mutant FBXW7-driven regorafenib resistance and contribute to the development of precision therapies to improve CRC treatment (Song et al., 2020). Similar findings have been observed for NSCLC (Ye et al., 2017). One study has demonstrated that MCL-1-binding BH3 mimetics or enhanced PUMA expression can overcome treatment resistance caused by MCL-1 stabilization in CRC cells (Tong et al., 2017). Several chemical classes of MCL-1 inhibitors have been developed (Abulwerdi et al., 2014; Nguyen et al., 2007; Kotschy et al., 2016). However existing MCL-1 inhibitors lack sufficient potency and specificity for cancer treatment. There is an urgent need for developing new MCL-1 inhibitors. These findings suggest that FBXW7 downstream oncoproteins, including MCL-1, are attractive targets for improving the efficacy of anticancer therapies.

### 6.3 Targeting FBXW7 to deplete CSCs

Tumor stem cells (CSCs) are a class of cell populations that are maintained in a non-proliferative state (referred to as quiescence, dormancy, or the G0 phase) and are a source of subsequent tumor recurrence and drug resistance (Eid et al., 2023). As mentioned previously, FBXW7 triggers drug resistance by triggering cell cycle arrest and quiescence in CSCs (Onoyama and Nakayama, 2008).Thus, the tumor-suppressive effect of FBXW7 protects CSCs from drug killing. It may be possible to sensitize CSCs to anticancer therapy by inducing their entry into the cell cycle via FBXW7 silencing (Takeishi and Nakayama, 2016). Studies have shown that FBXW7-deficient leukemia-initiating cells (LICs) elevates the levels of c-Myc and are more sensitive to the anti-cancer drug imatinib (Takeishi et al., 2013). This revealed that silencing FBXW7 leads to the loss of CML LIC self-renewal, which may represent a promising therapeutic approach for CML-resistant patients (Reavie et al., 2013). Another study suggested that inhibition of FBXW7 expression after chemotherapy is an attractive strategy for eradicating colorectal CSCs and may enhance their response to anticancer drugs (Izumi et al., 2017). Disseminated tumor cells (DTCs) are present in the bone marrow of patients with primary breast cancer, and their low proliferative state is a major cause of drug resistance (Hartkopf et al., 2015). In a mouse model of breast cancer, the ablation of FBXW7 awakened the proliferation of disseminated tumor cells (DTCs) and their sensitivity to paclitaxel treatment (Shimizu et al., 2019; Chen et al., 2023b). These studies suggest that promoting the re-entry of CSCs into the cell cycle may be an effective way to enhance the sensitivity of cancer therapies. However, the development of FBXW7 inhibitors is still in its infancy, and their long-term effects remain unclear.

### 6.4 Limitations and challenges of cancer treatment through FBXW7

Although many studies have proposed FBXW7 as a therapeutic target for cancer, no suitable drug has entered clinical studies to date (Fan et al., 2022). This suggests that targeting FBXW7 in cancer treatment poses significant obstacles. The intricate network of relationships involving numerous upstream regulators and downstream target proteins of FBXW7 presents a formidable hurdle. In most cases, resistance to one type of anticancer drug has not been fully elucidated as a singular pathway. Therefore, it is difficult to achieve the goal of overcoming cancer drug resistance by adjusting a single factor or target protein. For example, in addition to FBXW7, MCL1 is regulated by β-TrCP, MULE, and FBXO4 (Ding et al., 2007; Senichkin et al., 2020). The FBXW7 ubiquitination-degradation network usually involves multiple oncoproteins in different pathways that affect different biological functions. Therefore, before using FBXW7 as a therapeutic target, it is necessary to ensure that the intervention does not have any unintended consequences. In the future, it may be necessary to develop drugs with multi-pathway co-targeting or to identify the most critical factors that mediate resistance. In addition, the strategy of silencing FBXW7, a recognized tumor suppressor, can break the dormant state of CSCs. This is inevitably accompanied by concerns about whether it will promote cancer progression and metastasis at other sites. It is worth considering how the destructive effects of CSCs are caused by silencing FBXW7 and how its own tumor suppressor effects can be balanced. Although no drugs targeting FBXW7 have yet been approved for clinical use, several promising candidates deserve further evaluation.

## 7 Discussion

FBXW7 acts as a recognition component of the E3 ubiquitin ligase and mediates the ubiquitination and degradation of several proteins *in vivo*. An extensive protein regulatory network centered on FBXW7 plays a crucial role in the development of anticancer therapeutic resistance.

In this study, we review the involvement of imbalanced FBXW7 in the development of therapeutic resistance in cancer. Aberrant FBXW7 is involved in the development of cancer drug resistance (apoptosis resistance, EMT, stem cell characterization, shaping of the tumor microenvironment, autophagy and immune evasion) by causing an imbalance in oncoproteins. However, the FBXW7 regulatory network is complex, and it is clear that additional upstream or downstream factors are involved in the development of therapeutic resistance. Moreover, FBXW7 may also be involved in other mechanisms of tumor resistance development (e.g., enhanced DNA repair, drug target alteration, epigenetic alteration, promotion of drug metabolism and detoxification). The mechanism underlying FBXW7-mediated resistance or susceptibility must be elucidated to provide ideas for the subsequent eradication of resistance.

We also reviewed the effect of FBXW7 on the resistance to anticancer drugs (e.g., cisplatin, gefitinib, and pembrolizumab) in various human tumors. From chemotherapeutic drugs to targeted drugs and immunotherapeutic drugs, FBXW7 mutations or deletions adversely affect the therapeutic efficacy in all but a few tumors, namely, hematological tumors. Therefore, the mutational status of FBXW7 could serve as a suitable diagnostic biomarker and play an invaluable role in individualized cancer treatment. Next-generation sequencing (NGS) technology can broadly identify genetic differences in tumor tissue, which provides useful support for detecting many genetic changes associated with FBXW7 (Churi et al., 2014; Nemecek et al., 2016) (PMID: 25536104, 25536104).

Clinical evidence linking FBXW7 to resistance to multiple drug classes further suggests that this gene is a potential therapeutic target and prognostic biomarker for tumors. Development of drugs that modulate regulatory factors upstream of FBXW7 and substrates downstream of FBXW7 for use in cancer cells with FBXW7 inactivation can be considered. Notably, FBXW7 has a complex network of upstream and downstream relationships, and it is difficult to inhibit or activate a target alone to achieve its intended purpose. Future studies are needed to determine which is more beneficial for cancer treatment, whether by targeting a key factor in the FBXW7 network alone or by using combination therapies that target the upstream, downstream, or parallel pathways of FBXW7. In addition, therapies that inactivate FBXW7 and allow tumor stem cells to enter the cell cycle should be considered with caution. Therefore, there is a need to develop fine-tuned drug-specific therapies for specific tumors by weighing the tumor-suppressive effects of FBXW7 against increased drug sensitivity.

In conclusion, FBXW7 plays a crucial role in the development of anticancer drug resistance. This review suggests new strategies for developing novel targeted interventions and enhancing tumor cell sensitivity to cancer therapies.

## Figures and Tables

**Table:** 

**UPS**	Ubiquitin-proteasome system
**FBXW7**	F-box and WD Repeat Domain-containing-7
**TME**	tumor microenvironment
**CSCs**	cancer stem cells
**EMT**	epithelial-mesenchymal transition
**AKT**	V-akt murine thymoma viral oncogene homolog
**Bcl-2**	B-cell lymphoma 2
**mTOR**	Mammalian target of rapamycin
**MAPK**	mitogen-activated protein kinases
**IGF-1**	Insulin-like growth factor-1
**NF-κB**	nuclear factor-kappa
**SCF E3 ligase**	KP1-CUL1-F box protein E3 ubiquitin ligases
**ESCC**	esophageal squamous cell carcinoma
**KLF5**	Krüppel-like factor-5
**C/EBPδ**	CCAAT/enhancer binding protein delta
**Cyclin E**	E-type cyclins
**iCCA**	intrahepatic cholangiocarcinoma
**ATL**	Adult T-cell leukemia-lymphoma
**CLL**	Chronic lymphocytic leukemia
**APC**	adenomatous polyposis coli
**T-ALL**	Adult “T cell” acute lymphoblastic leukemia
**MCL-1**	Myeloid leukemia 1
**MULE**	HECT domain-containing ubiquitin E3 ligase
**β-TrCP**	beta-transducin repeats-containing proteins
**ZEB1**	Zinc finger E-box binding homeobox 1
**LICs**	leukemia-initiating cells MiRNA: MicroRNA
**CAFs**	Cancer associated fibroblasts
**PD-1/PD-L1**	Programmed cell death protein 1/programmed cell death ligand 1
**EYA2**	Eyes absent homolog 2
**ICBs**	Immune-checkpoint blockers
**NSCLC**	Non-Small Cell Lung Cancer
**CRC**	Colorectal Cancer
**GC**	gastric cancer
**EGFR**	epidermal growth factor receptor
**HCC**	hepatocellular carcinoma
**PC**	pancreatic cancer
**TMZ**	temozolomide
**NPC**	nasopharyngeal carcinoma
**NFAT1**	nuclear factor of activated T-cells
**CML**	Chronic myeloid leukemia
**CRY2**	cryptochrome 2
**Hes-5**	Hairy and Enhancer-of-split homologues 5

## References

[B1] Abulwerdi F. Liao C. Liu M. Azmi A. S. Aboukameel A. Mady A. S. A. (2014). A novel small-molecule inhibitor of mcl-1 blocks pancreatic cancer growth *in vitro* and *in vivo* . Mol. Cancer Ther. 13 (3), 565–575. 10.1158/1535-7163.MCT-12-0767 24019208 PMC4174574

[B2] Akhoondi S. Lindström L. Widschwendter M. Corcoran M. Bergh J. Spruck C. (2010). Inactivation of FBXW7/hCDC4-β expression by promoter hypermethylation is associated with favorable prognosis in primary breast cancer. Breast Cancer Res. 12 (6), R105. 10.1186/bcr2788 21122106 PMC3046450

[B3] Akhoondi S. Sun D. von der Lehr N. Apostolidou S. Klotz K. Maljukova A. (2007). FBXW7/hCDC4 is a general tumor suppressor in human cancer. Cancer Res. 67 (19), 9006–9012. 10.1158/0008-5472.CAN-07-1320 17909001

[B4] Assaraf Y. G. Brozovic A. Gonçalves A. C. Jurkovicova D. Linē A. Machuqueiro M. (2019). The multi-factorial nature of clinical multidrug resistance in cancer. Drug Resist Updat 46, 100645. 10.1016/j.drup.2019.100645 31585396

[B5] Aydin I. T. Melamed R. D. Adams S. J. Castillo-Martin M. Demir A. Bryk D. (2014). FBXW7 mutations in melanoma and a new therapeutic paradigm. J. Natl. Cancer Inst. 106 (6), dju107. 10.1093/jnci/dju107 24838835 PMC4081626

[B6] Babaei-Jadidi R. Li N. Saadeddin A. Spencer-Dene B. Jandke A. Muhammad B. (2011). FBXW7 influences murine intestinal homeostasis and cancer, targeting Notch, Jun, and DEK for degradation. J. Exp. Med. 208 (2), 295–312. 10.1084/jem.20100830 21282377 PMC3039859

[B7] Bailey S. T. Miron P. L. Choi Y. J. Kochupurakkal B. Maulik G. Rodig S. J. (2014). NF-κB activation-induced anti-apoptosis renders HER2-positive cells drug resistant and accelerates tumor growth. Mol. Cancer Res. 12 (3), 408–420. 10.1158/1541-7786.MCR-13-0206-T 24319068 PMC4026253

[B8] Balamurugan K. Sharan S. Klarmann K. D. Zhang Y. Coppola V. Summers G. H. (2013). FBXW7α attenuates inflammatory signalling by downregulating C/EBPδ and its target gene Tlr4. Nat. Commun. 4, 1662. 10.1038/ncomms2677 23575666 PMC3625980

[B9] Balamurugan K. Sterneck E. (2013). The many faces of C/EBPδ and their relevance for inflammation and cancer. Int. J. Biol. Sci. 9 (9), 917–933. 10.7150/ijbs.7224 24155666 PMC3805898

[B10] Bayik D. Lathia J. D. (2021). Cancer stem cell-immune cell crosstalk in tumour progression. Nat. Rev. Cancer 21 (8), 526–536. 10.1038/s41568-021-00366-w 34103704 PMC8740903

[B11] Berndsen C. E. Wolberger C. (2014). New insights into ubiquitin E3 ligase mechanism. Nat. Struct. Mol. Biol. 21 (4), 301–307. 10.1038/nsmb.2780 24699078

[B12] Boutilier A. J. Elsawa S. F. (2021). Macrophage polarization states in the tumor microenvironment. Int. J. Mol. Sci. 22 (13), 6995. 10.3390/ijms22136995 34209703 PMC8268869

[B13] Bray F. Laversanne M. Weiderpass E. Soerjomataram I. (2021). The ever-increasing importance of cancer as a leading cause of premature death worldwide. Cancer 127 (16), 3029–3030. 10.1002/cncr.33587 34086348

[B14] Cao J. Ge M. H. Ling Z. Q. (2016). Fbxw7 tumor suppressor: a vital regulator contributes to human tumorigenesis. Med. Baltim. 95 (7), e2496. 10.1097/MD.0000000000002496 PMC4998596 26886596

[B15] Chang H. Zou Z. (2020). Targeting autophagy to overcome drug resistance: further developments. J. Hematol. Oncol. 13 (1), 159. 10.1186/s13045-020-01000-2 33239065 PMC7687716

[B16] Chen S. Leng P. Guo J. Zhou H. (2023b). FBXW7 in breast cancer: mechanism of action and therapeutic potential. J. Exp. Clin. Cancer Res. 42, 226. 10.1186/s13046-023-02767-1 37658431 PMC10474666

[B17] Chen S. Lin J. Zhao J. Lin Q. Liu J. Wang Q. (2023a). FBXW7 attenuates tumor drug resistance and enhances the efficacy of immunotherapy. Front. Oncol. 13, 1147239. 10.3389/fonc.2023.1147239 36998461 PMC10043335

[B18] Cheng Y. Li G. (2012). Role of the ubiquitin ligase Fbw7 in cancer progression. Cancer Metastasis Rev. 31 (1–2), 75–87. 10.1007/s10555-011-9330-z 22124735

[B19] Cheok C. F. Verma C. S. Baselga J. Lane D. P. (2011). Translating p53 into the clinic. Nat. Rev. Clin. Oncol. 8 (1), 25–37. 10.1038/nrclinonc.2010.174 20975744

[B20] Cho K. B. Cho M. K. Lee W. Y. Kang K. W. (2010). Overexpression of c-myc induces epithelial mesenchymal transition in mammary epithelial cells. Cancer Lett. 293 (2), 230–239. 10.1016/j.canlet.2010.01.013 20144848

[B21] Churi C. R. Shroff R. Wang Y. Rashid A. Kang H. C. Weatherly J. (2014). Mutation profiling in cholangiocarcinoma: prognostic and therapeutic implications. PLoS One 9 (12), e115383. 10.1371/journal.pone.0115383 25536104 PMC4275227

[B22] Close V. Close W. Kugler S. J. Reichenzeller M. Yosifov D. Y. Bloehdorn J. (2019). FBXW7 mutations reduce binding of NOTCH1, leading to cleaved NOTCH1 accumulation and target gene activation in CLL. Blood 133 (8), 830–839. 10.1182/blood-2018-09-874529 30510140

[B23] Cremona C. A. Sancho R. Diefenbacher M. E. Behrens A. (2016). Fbw7 and its counteracting forces in stem cells and cancer: oncoproteins in the balance. Semin. Cancer Biol. 36, 52–61. 10.1016/j.semcancer.2015.09.006 26410034

[B24] Cuevas I. C. Sahoo S. S. Kumar A. Zhang H. Westcott J. Aguilar M. (2019). Fbxw7 is a driver of uterine carcinosarcoma by promoting epithelial-mesenchymal transition. Proc. Natl. Acad. Sci. U. S. A. 116 (51), 25880–25890. 10.1073/pnas.1911310116 31772025 PMC6926017

[B25] Ding J. Zhao Z. Song J. Luo B. Huang L. (2018). MiR-223 promotes the doxorubicin resistance of colorectal cancer cells via regulating epithelial-mesenchymal transition by targeting FBXW7. Acta Biochim. Biophys. Sin. (Shanghai). 50 (6), 597–604. 10.1093/abbs/gmy040 29701752

[B26] Davis R. J. Welcker M. Clurman B. E. (2014). Tumor suppression by the Fbw7 ubiquitin ligase: mechanisms and opportunities. Cancer Cell 26 (4), 455–464. 10.1016/j.ccell.2014.09.013 25314076 PMC4227608

[B27] Díaz V. M. de Herreros A. G. (2016). F-box proteins: keeping the epithelial-to-mesenchymal transition (EMT) in check. Semin. Cancer Biol. 36, 71–79. 10.1016/j.semcancer.2015.10.003 26506454

[B28] Ding P. Ma Z. Fan Y. Feng Y. Shao C. Pan M. (2023). Emerging role of ubiquitination/deubiquitination modification of PD-1/PD-L1 in cancer immunotherapy. Genes Dis. 10 (3), 848–863. 10.1016/j.gendis.2022.01.002 37396527 PMC10308071

[B29] Ding Q. He X. Hsu J. M. Xia W. Chen C. T. Li L. Y. (2007). Degradation of Mcl-1 by beta-TrCP mediates glycogen synthase kinase 3-induced tumor suppression and chemosensitization. Mol. Cell Biol. 27 (11), 4006–4017. 10.1128/MCB.00620-06 17387146 PMC1900029

[B30] Dongre A. Weinberg R. A. (2019). New insights into the mechanisms of epithelial-mesenchymal transition and implications for cancer. Nat. Rev. Mol. Cell Biol. 20 (2), 69–84. 10.1038/s41580-018-0080-4 30459476

[B31] Duffy M. J. Synnott N. C. O’Grady S. Crown J. (2022). Targeting p53 for the treatment of cancer. Semin. Cancer Biol. 79, 58–67. 10.1016/j.semcancer.2020.07.005 32741700

[B32] Eid R. A. Alaa Edeen M. Shedid E. M. Kamal A. S. S. Warda M. M. Mamdouh F. (2023). Targeting cancer stem cells as the key driver of carcinogenesis and therapeutic resistance. Int. J. Mol. Sci. 24 (2), 1786. 10.3390/ijms24021786 36675306 PMC9861138

[B33] Elizabeth Caldon C. (2020). Friends and foes: our evolving understanding of the link between Fbxw7 and p53 in cancer. Neoplasia 22 (11), 659–660. 10.1016/j.neo.2020.07.007 33070871 PMC7573499

[B34] Erin N. Grahovac J. Brozovic A. Efferth T. (2020). Tumor microenvironment and epithelial mesenchymal transition as targets to overcome tumor multidrug resistance. Drug Resist Updat 53, 100715. 10.1016/j.drup.2020.100715 32679188

[B35] Essers M. A. G. Trumpp A. (2010). Targeting leukemic stem cells by breaking their dormancy. Mol. Oncol. 4 (5), 443–450. 10.1016/j.molonc.2010.06.001 20599449 PMC5527930

[B36] Essmann F. Schulze-Osthoff K. (2012). Translational approaches targeting the p53 pathway for anti-cancer therapy. Br. J. Pharmacol. 165 (2), 328–344. 10.1111/j.1476-5381.2011.01570.x 21718309 PMC3268188

[B37] Eto K. Iwatsuki M. Watanabe M. Ishimoto T. Ida S. Imamura Y. The sensitivity of gastric cancer to trastuzumab is regulated by the miR-223/FBXW7 pathway. Int. J. Cancer. 136 (7), 1537–45. 10.1002/ijc.29168 25159729

[B38] Fan J. Bellon M. Ju M. Zhao L. Wei M. Fu L. (2022). Clinical significance of FBXW7 loss of function in human cancers. Mol. Cancer 21 (1), 87. 10.1186/s12943-022-01548-2 35346215 PMC8962602

[B39] Fang L. Yang Z. Zhou J. Tung J. Y. Hsiao C. D. Wang L. (2015). Circadian clock gene CRY2 degradation is involved in chemoresistance of colorectal cancer. Mol. Cancer Ther. 14 (6), 1476–1487. 10.1158/1535-7163.MCT-15-0030 25855785 PMC4458447

[B40] Fender A. W. Nutter J. M. Fitzgerald T. L. Bertrand F. E. Sigounas G. (2015). Notch-1 promotes stemness and epithelial to mesenchymal transition in colorectal cancer. J. Cell Biochem. 116 (11), 2517–2527. 10.1002/jcb.25196 25914224

[B41] Feng X. Zou B. Nan T. Zheng X. Zheng L. Lan J. (2022). MiR-25 enhances autophagy and promotes sorafenib resistance of hepatocellular carcinoma via targeting FBXW7. Int. J. Med. Sci. 19 (2), 257–266. 10.7150/ijms.67352 35165511 PMC8795798

[B42] Forbes S. A. Beare D. Boutselakis H. Bamford S. Bindal N. Tate J. (2017). COSMIC: somatic cancer genetics at high-resolution. Nucleic Acids Res. 45 (D1), D777–83. 10.1093/nar/gkw1121 27899578 PMC5210583

[B43] Galindo-Moreno M. Giráldez S. Limón-Mortés M. C. Belmonte-Fernández A. Sáez C. Japón M. Á. (2020). p53 and FBXW7: sometimes two guardians are worse than one. Cancers (Basel) 12 (4), 985. 10.3390/cancers12040985 32316282 PMC7225930

[B44] Gallyas F. Sumegi B. Szabo C. (2020). Role of akt activation in PARP inhibitor resistance in cancer. Cancers (Basel) 12 (3), 532. 10.3390/cancers12030532 32106627 PMC7139751

[B45] Gong J. Zhou Y. Liu D. Huo J. (2018). F-box proteins involved in cancer-associated drug resistance. Oncol. Lett. 15 (6), 8891–8900. 10.3892/ol.2018.8500 29805625 PMC5958692

[B46] Gstalder C. Liu D. Miao D. Lutterbach B. DeVine A. L. Lin C. (2020). Inactivation of Fbxw7 impairs dsRNA sensing and confers resistance to PD-1 blockade. Cancer Discov. 10 (9):1296–311. 32371478 10.1158/2159-8290.CD-19-1416 PMC8802534

[B47] Guo G. Yu M. Xiao W. Celis E. Cui Y. (2017). Local activation of p53 in the tumor microenvironment overcomes immune suppression and enhances antitumor immunity. Cancer Res. 77 (9), 2292–2305. 10.1158/0008-5472.CAN-16-2832 28280037 PMC5465961

[B48] Gupta A. Shah K. Oza M. J. Behl T. (2019). Reactivation of p53 gene by MDM2 inhibitors: a novel therapy for cancer treatment. Biomed. Pharmacother. 109, 484–492. 10.1016/j.biopha.2018.10.155 30551517

[B49] Hales E. C. Taub J. W. Matherly L. H. (2014). New insights into Notch1 regulation of the PI3K-AKT-mTOR1 signaling axis: targeted therapy of γ-secretase inhibitor resistant T-cell acute lymphoblastic leukemia. Cell Signal 26 (1), 149–161. 10.1016/j.cellsig.2013.09.021 24140475

[B50] Hartkopf A. D. Wallwiener M. Fehm T. N. Hahn M. Walter C. B. Gruber I. (2015). Disseminated tumor cells from the bone marrow of patients with nonmetastatic primary breast cancer are predictive of locoregional relapse. Ann. Oncol. 26 (6), 1155–1160. 10.1093/annonc/mdv148 25791636

[B51] Hou Y. C. Deng J. Y. (2015). Role of E3 ubiquitin ligases in gastric cancer. World J. Gastroenterol. 21 (3), 786–793. 10.3748/wjg.v21.i3.786 25624711 PMC4299330

[B52] Hu J. L. Wang W. Lan X. L. Zeng Z. C. Liang Y. S. Yan Y. R. (2019). CAFs secreted exosomes promote metastasis and chemotherapy resistance by enhancing cell stemness and epithelial-mesenchymal transition in colorectal cancer. Mol. Cancer 18 (1), 91. 10.1186/s12943-019-1019-x 31064356 PMC6503554

[B53] Hu X. Wen L. Li X. Zhu C. (2023). Relationship between autophagy and drug resistance in tumors. Mini Rev. Med. Chem. 23 (10), 1072–1078. 10.2174/1389557522666220905090732 36065919

[B54] Huang Y. Hong W. Wei X. (2022). The molecular mechanisms and therapeutic strategies of EMT in tumor progression and metastasis. J. Hematol. Oncol. 15 (1), 129. 10.1186/s13045-022-01347-8 36076302 PMC9461252

[B55] Hughes R. Qian B. Z. Rowan C. Muthana M. Keklikoglou I. Olson O. C. (2015). Perivascular M2 macrophages stimulate tumor relapse after chemotherapy. Cancer Res. 75 (17), 3479–3491. 10.1158/0008-5472.CAN-14-3587 26269531 PMC5024531

[B56] Inuzuka H. Shaik S. Onoyama I. Gao D. Tseng A. Maser R. S. (2011). SCF(FBW7) regulates cellular apoptosis by targeting MCL1 for ubiquitylation and destruction. Nature 471 (7336), 104–109. 10.1038/nature09732 21368833 PMC3076007

[B57] Iorio M. V. Croce C. M. (2012). MicroRNA dysregulation in cancer: diagnostics, monitoring and therapeutics. A comprehensive review. EMBO Mol. Med. 4 (3), 143–159. 10.1002/emmm.201100209 22351564 PMC3376845

[B58] Iraz A. T. Abbate F. Geena S. R. Badal B. Iannis A. Desman G. (2017). FBXW7 inactivation in a BrafV600E -driven mouse model leads to melanoma development. Pigment Cell & melanoma Res. 30 (6), 571–574. 10.1111/pcmr.12603 28581198 PMC5668175

[B59] Ishii N. Araki K. Yokobori T. Gantumur D. Yamanaka T. Altan B. (2017). Reduced FBXW7 expression in pancreatic cancer correlates with poor prognosis and chemotherapeutic resistance via accumulation of MCL1. Oncotarget 8 (68), 112636–112646. 10.18632/oncotarget.22634 29348852 PMC5762537

[B60] Izumi D. Ishimoto T. Miyake K. Eto T. Arima K. Kiyozumi Y. (2017). Colorectal cancer stem cells acquire chemoresistance through the upregulation of F-box/WD repeat-containing protein 7 and the consequent degradation of c-myc. Stem Cells 35 (9), 2027–2036. 10.1002/stem.2668 28699179

[B61] Jiang Y. Song Y. Wang R. Hu T. Zhang D. Wang Z. (2019). NFAT1-Mediated regulation of NDEL1 promotes growth and invasion of glioma stem-like cells. Cancer Res. 79 (10), 2593–2603. 10.1158/0008-5472.CAN-18-3297 30940662

[B62] Jiménez-Izquierdo R. Morrugares R. Suanes-Cobos L. Correa-Sáez A. Garrido-Rodríguez M. Cerero-Tejero L. (2023). FBXW7 tumor suppressor regulation by dualspecificity tyrosine-regulated kinase 2. Cell Death Dis. 14 (3), 202. 10.1038/s41419-023-05724-0 36934104 PMC10024693

[B63] Kapoor I. Bodo J. Hill B. T. Hsi E. D. Almasan A. (2020). Targeting BCL-2 in B-cell malignancies and overcoming therapeutic resistance. Cell Death Dis. 11 (11), 941. 10.1038/s41419-020-03144-y 33139702 PMC7608616

[B64] Kim Y. C. Guan K. L. (2015). mTOR: a pharmacologic target for autophagy regulation. J. Clin. Invest. 125 (1), 25–32. 10.1172/JCI73939 25654547 PMC4382265

[B65] King B. Trimarchi T. Reavie L. Xu L. Mullenders J. Ntziachristos P. (2013). The ubiquitin ligase FBXW7 modulates leukemia-initiating cell activity by regulating MYC stability. Cell 153 (7), 1552–1566. 10.1016/j.cell.2013.05.041 23791182 PMC4146439

[B66] Kipreos E. T. Pagano M. (2000). The F-box protein family. Genome Biol. 1 (5), REVIEWS3002. 10.1186/gb-2000-1-5-reviews3002 11178263 PMC138887

[B67] Koepp D. M. Schaefer L. K. Ye X. Keyomarsi K. Chu C. Harper J. W. (2001). Phosphorylation-dependent ubiquitination of cyclin E by the SCFFbw7 ubiquitin ligase. Science 294 (5540), 173–177. 10.1126/science.1065203 11533444

[B68] Kothari N. Teer J. K. Abbott A. M. Srikumar T. Zhang Y. Yoder S. J. (2016). Increased incidence of FBXW7 and POLE proofreading domain mutations in young adult colorectal cancers. Cancer 122 (18), 2828–2835. 10.1002/cncr.30082 27244218 PMC5014625

[B69] Kotschy A. Szlavik Z. Murray J. Davidson J. Maragno A. L. Le Toumelin-Braizat G. (2016). The MCL1 inhibitor S63845 is tolerable and effective in diverse cancer models. Nature 538 (7626), 477–482. 10.1038/nature19830 27760111

[B70] Kraszewska M. D. Dawidowska M. Kosmalska M. Sędek L. Grzeszczak W. Kowalczyk J. R. (2013). BCL11B, FLT3, NOTCH1 and FBXW7 mutation status in T-cell acute lymphoblastic leukemia patients. Blood Cells Mol. Dis. 50 (1), 33–38. 10.1016/j.bcmd.2012.09.001 23040356

[B71] Krishna B. M. Jana S. Singhal J. Horne D. Awasthi S. Salgia R. (2019). Notch signaling in breast cancer: from pathway analysis to therapy. Cancer Lett. 461, 123–131. 10.1016/j.canlet.2019.07.012 31326555 PMC9003668

[B72] Kumar A. Singh U. K. Chaudhary A. (2015). Targeting autophagy to overcome drug resistance in cancer therapy. Future Med. Chem. 7 (12), 1535–1542. 10.4155/fmc.15.88 26334206

[B73] Kumar V. Palermo R. Talora C. Campese A. F. Checquolo S. Bellavia D. (2014). Notch and NF-kB signaling pathways regulate miR-223/FBXW7 axis in T-cell acute lymphoblastic leukemia. Leukemia 28 (12), 2324–2335. 10.1038/leu.2014.133 24727676

[B74] Lamouille S. Xu J. Derynck R. (2014). Molecular mechanisms of epithelial-mesenchymal transition. Nat. Rev. Mol. Cell Biol. 15 (3), 178–196. 10.1038/nrm3758 24556840 PMC4240281

[B75] Lan H. Sun Y. (2019). FBXW7 E3 ubiquitin ligase: degrading, not degrading, or being degraded. Protein Cell 10 (12), 861–863. 10.1007/s13238-019-0652-x 31342282 PMC6881281

[B76] Lee S. Rauch J. Kolch W. (2020a). Targeting MAPK signaling in cancer: mechanisms of drug resistance and sensitivity. Int. J. Mol. Sci. 21 (3), 1102. 10.3390/ijms21031102 32046099 PMC7037308

[B77] Lee Y. S. Song S. J. Hong H. K. Oh B. Y. Lee W. Y. Cho Y. B. (2020b). The FBW7-MCL-1 axis is key in M1 and M2 macrophage-related colon cancer cell progression: validating the immunotherapeutic value of targeting PI3Kγ. Exp. Mol. Med. 52 (5), 815–831. 10.1038/s12276-020-0436-7 32444799 PMC7272616

[B78] Li H. Wang Z. Zhang W. Qian K. Xu W. Zhang S. (2016b). Fbxw7 regulates tumor apoptosis, growth arrest and the epithelial-to-mesenchymal transition in part through the RhoA signaling pathway in gastric cancer. Cancer Lett. 370 (1), 39–55. 10.1016/j.canlet.2015.10.006 26458995

[B79] Li N. Babaei-Jadidi R. Lorenzi F. Spencer-Dene B. Clarke P. Domingo E. (2019). An FBXW7-ZEB2 axis links EMT and tumour microenvironment to promote colorectal cancer stem cells and chemoresistance. Oncogenesis 8 (3), 13. 10.1038/s41389-019-0125-3 30783098 PMC6381143

[B80] Li N. Lorenzi F. Kalakouti E. Normatova M. Babaei-Jadidi R. Tomlinson I. (2015). FBXW7-mutated colorectal cancer cells exhibit aberrant expression of phosphorylated-p53 at Serine-15. Oncotarget 6 (11), 9240–9256. 10.18632/oncotarget.3284 25860929 PMC4496214

[B81] Li W. Zhang H. Assaraf Y. G. Zhao K. Xu X. Xie J. (2016a). Overcoming ABC transporter-mediated multidrug resistance: molecular mechanisms and novel therapeutic drug strategies. Drug Resist Updat 27, 14–29. 10.1016/j.drup.2016.05.001 27449595

[B82] Li Y. Wang Z. Ajani J. A. Song S. (2021). Drug resistance and Cancer stem cells. Cell Commun. Signal 19 (1), 19. 10.1186/s12964-020-00627-5 33588867 PMC7885480

[B83] Lilienbaum A. (2013). Relationship between the proteasomal system and autophagy. Int. J. Biochem. Mol. Biol. 4 (1), 1–26. 23638318 PMC3627065

[B84] Lin J. Ji A. Qiu G. Feng H. Li J. Li S. (2018). FBW7 is associated with prognosis, inhibits malignancies and enhances temozolomide sensitivity in glioblastoma cells. Cancer Sci. 109 (4), 1001–1011. 10.1111/cas.13528 29427543 PMC5891203

[B85] Lin L. Ding D. Xiao X. Li B. Cao P. Li S. (2020). Trametinib potentiates TRAIL-induced apoptosis via FBW7-dependent Mcl-1 degradation in colorectal cancer cells. J. Cell Mol. Med. 24 (12), 6822–6832. 10.1111/jcmm.15336 32352219 PMC7299726

[B86] Lin M. Xu Y. Gao Y. Pan C. Zhu X. Wang Z. W. (2019). Regulation of F-box proteins by noncoding RNAs in human cancers. Cancer Lett. 466, 61–70. 10.1016/j.canlet.2019.09.008 31546023

[B87] Lin P. C. Yeh Y. M. Hsu H. P. Chan R. H. Lin B. W. Chen P. C. (2021). Comprehensively exploring the mutational landscape and patterns of genomic evolution in hypermutated cancers. Cancers (Basel) 13 (17), 4317. 10.3390/cancers13174317 34503126 PMC8431047

[B88] Liu H. Wang K. Fu H. Song J. (2018). Low expression of the ubiquitin ligase FBXW7 correlates with poor prognosis of patients with colorectal cancer. Int. J. Clin. Exp. Pathol. 11 (1), 413–419. 31938126 PMC6957979

[B89] Liu H. Zhao H. Sun Y. (2022a). Tumor microenvironment and cellular senescence: understanding therapeutic resistance and harnessing strategies. Semin. Cancer Biol. 86 (3), 769–781. 10.1016/j.semcancer.2021.11.004 34799201

[B90] Liu J. Wei L. Hu N. Wang D. Ni J. Zhang S. (2022b). FBW7-mediated ubiquitination and destruction of PD-1 protein primes sensitivity to anti-PD-1 immunotherapy in non-small cell lung cancer. J. Immunother. Cancer 10 (9), e005116. 10.1136/jitc-2022-005116 36104103 PMC9476142

[B91] Liu N. Li H. Li S. Shen M. Xiao N. Chen Y. (2010). The Fbw7/human CDC4 tumor suppressor targets proproliferative factor KLF5 for ubiquitination and degradation through multiple phosphodegron motifs. J. Biol. Chem. 285 (24), 18858–18867. 10.1074/jbc.M109.099440 20388706 PMC2881808

[B92] Liu Q. Aminu B. Roscow O. Zhang W. (2021). Targeting the ubiquitin signaling cascade in tumor microenvironment for cancer therapy. Int. J. Mol. Sci. 22 (2), 791. 10.3390/ijms22020791 33466790 PMC7830467

[B93] Liu W. Ren D. Xiong W. Jin X. Zhu L. (2022c). A novel FBW7/NFAT1 axis regulates cancer immunity in sunitinib-resistant renal cancer by inducing PD-L1 expression. J. Exp. Clin. Cancer Res. 41 (1), 38. 10.1186/s13046-022-02253-0 35081978 PMC8790872

[B94] Lixia W. Xiantao Y. Yueyong L. Wenyi W. Wang Z. (2014). Aberrant regulation of FBW7 in cancer. Oncotarget 5 (8), 2000–2015. 10.18632/oncotarget.1859 24899581 PMC4039140

[B95] Lorenzi F. Babaei-Jadidi R. Sheard J. Spencer-Dene B. Nateri A. S. (2016). Fbxw7-associated drug resistance is reversed by induction of terminal differentiation in murine intestinal organoid culture. Mol. Ther. Methods Clin. Dev. 3, 16024. 10.1038/mtm.2016.24 27110583 PMC4830362

[B96] Lupini L. Bassi C. Mlcochova J. Musa G. Russo M. Vychytilova-Faltejskova P. (2015). Prediction of response to anti-EGFR antibody-based therapies by multigene sequencing in colorectal cancer patients. BMC Cancer 15, 808. 10.1186/s12885-015-1752-5 26508446 PMC4624582

[B97] Ma J. Cheng L. Liu H. Zhang J. Shi Y. Zeng F. (2013). Genistein down-regulates miR-223 expression in pancreatic cancer cells. Curr. Drug Targets 14 (10), 1150–1156. 10.2174/13894501113149990187 23834147

[B98] Malyukova A. Brown S. Papa R. O’Brien R. Giles J. Trahair T. N. (2013). FBXW7 regulates glucocorticoid response in T-cell acute lymphoblastic leukaemia by targeting the glucocorticoid receptor for degradation. Leukemia 27 (5), 1053–1062. 10.1038/leu.2012.361 23228967

[B99] Mansoori B. Mohammadi A. Davudian S. Shirjang S. Baradaran B. (2017). The different mechanisms of cancer drug resistance: a brief review. Adv. Pharm. Bull. 7 (3), 339–348. 10.15171/apb.2017.041 29071215 PMC5651054

[B100] Mao J. H. Perez-Losada J. Wu D. Delrosario R. Tsunematsu R. Nakayama K. I. (2004). Fbxw7/Cdc4 is a p53-dependent, haploinsufficient tumour suppressor gene. Nature 432, 775–779. 10.1038/nature03155 15592418

[B101] Matsumoto A. Tateishi Y. Onoyama I. Okita Y. Nakayama K. Nakayama K. I. (2011). Fbxw7β resides in the endoplasmic reticulum membrane and protects cells from oxidative stress. Cancer Sci. 102 (4), 749–755. 10.1111/j.1349-7006.2011.01851.x 21205095

[B102] Mihashi Y. Mizoguchi M. Takamatsu Y. Ishitsuka K. Iwasaki H. Koga M. (2017). C-MYC and its main ubiquitin ligase, FBXW7, influence cell proliferation and prognosis in adult T-cell leukemia/lymphoma. Am. J. Surg. Pathol. 41 (8), 1139–1149. 10.1097/PAS.0000000000000871 28498285

[B103] Mittal P. Singh S. Sinha R. Shrivastava A. Singh A. Singh I. K. (2021). Myeloid cell leukemia 1 (MCL-1): Structural characteristics and application in cancer therapy. Int. J. Biol. Macromol. 187, 999–1018. 10.1016/j.ijbiomac.2021.07.166 34339789

[B104] Mohammad R. M. Muqbil I. Lowe L. Yedjou C. Hsu H. Y. Lin L. T. (2015). Broad targeting of resistance to apoptosis in cancer. Semin. Cancer Biol. 35, S78–S103. 10.1016/j.semcancer.2015.03.001 25936818 PMC4720504

[B105] Murugan A. K. (2019). mTOR: role in cancer, metastasis and drug resistance. Semin. Cancer Biol. 59, 92–111. 10.1016/j.semcancer.2019.07.003 31408724

[B106] Nash P. Tang X. Orlicky S. Chen Q. Gertler F. B. Mendenhall M. D. (2001). Multisite phosphorylation of a CDK inhibitor sets a threshold for the onset of DNA replication. Nature 414 (6863), 514–521. 10.1038/35107009 11734846

[B107] Nemecek R. Berkovcova J. Radova L. Kazda T. Mlcochova J. Vychytilova-Faltejskova P. (2016). Mutational analysis of primary and metastatic colorectal cancer samples underlying the resistance to cetuximab-based therapy. Onco Targets Ther. 9, 4695–4703. 10.2147/OTT.S102891 27555788 PMC4968864

[B108] Nguyen M. Marcellus R. C. Roulston A. Watson M. Serfass L. Murthy Madiraju S. R. (2007). Small molecule obatoclax (GX15-070) antagonizes MCL-1 and overcomes MCL-1-mediated resistance to apoptosis. Proc. Natl. Acad. Sci. U. S. A. 104 (49), 19512–19517. 10.1073/pnas.0709443104 18040043 PMC2148320

[B109] Nussinov R. Tsai C. J. Jang H. (2021). Anticancer drug resistance: an update and perspective. Drug Resist Updat 59, 100796. 10.1016/j.drup.2021.100796 34953682 PMC8810687

[B110] O’Neil J. Grim J. Strack P. Rao S. Tibbitts D. Winter C. (2007). FBW7 mutations in leukemic cells mediate NOTCH pathway activation and resistance to gamma-secretase inhibitors. J. Exp. Med. 204 (8), 1813–1824. 10.1084/jem.20070876 17646409 PMC2118656

[B111] Onoyama I. Nakayama K. I. (2008). Fbxw7 in cell cycle exit and stem cell maintenance: insight from gene-targeted mice. Cell Cycle 7 (21), 3307–3313. 10.4161/cc.7.21.6931 18948752

[B112] Onoyama I. Tsunematsu R. Matsumoto A. Kimura T. de Alborán I. M. Nakayama K. (2007). Conditional inactivation of Fbxw7 impairs cell-cycle exit during T cell differentiation and results in lymphomatogenesis. J. Exp. Med. 204 (12), 2875–2888. 10.1084/jem.20062299 17984302 PMC2118521

[B113] Orlicky S. Tang X. Willems A. Tyers M. Sicheri F. (2003). Structural basis for phosphodependent substrate selection and orientation by the SCFCdc4 ubiquitin ligase. Cell 112 (2), 243–256. 10.1016/s0092-8674(03)00034-5 12553912

[B114] Pan Y. Liu J. Gao Y. Guo Y. Wang C. Liang Z. (2023). FBXW7 loss of function promotes esophageal squamous cell carcinoma progression via elevating MAP4 and ERK phosphorylation. J. Exp. Clin. Cancer Res. 42 (1), 75. 10.1186/s13046-023-02630-3 36991467 PMC10054043

[B115] Peled N. Wynes M. W. Ikeda N. Ohira T. Yoshida K. Qian J. (2013). Insulin-like growth factor-1 receptor (IGF-1R) as a biomarker for resistance to the tyrosine kinase inhibitor gefitinib in non-small cell lung cancer. Cell Oncol. (Dordr) 36 (4), 277–288. 10.1007/s13402-013-0133-9 23619944 PMC4186686

[B116] Qin Y. Ashrafizadeh M. Mongiardini V. Grimaldi B. Crea F. Rietdorf K. (2023). Autophagy and cancer drug resistance in dialogue: pre-clinical and clinical evidence. Cancer Lett. 570, 216307. 10.1016/j.canlet.2023.216307 37451426

[B117] Qiu B. Sun Y. Nie W. Yang Q. Guo X. (2023). FBXW7 promotes autophagy and inhibits proliferation of oral squamous cell carcinoma. Immun. Inflamm. Dis. 11 (5), e845. 10.1002/iid3.845 37249289 PMC10187000

[B118] Quinn B. A. Dash R. Azab B. Sarkar S. Das S. K. Kumar S. (2011). Targeting Mcl-1 for the therapy of cancer. Expert Opin. Investig. Drugs 20 (10), 1397–1411. 10.1517/13543784.2011.609167 PMC3205956 21851287

[B119] Ramos A. Sadeghi S. Tabatabaeian H. (2021). Battling chemoresistance in cancer: root causes and strategies to uproot them. Int. J. Mol. Sci. 22 (17), 9451. 10.3390/ijms22179451 34502361 PMC8430957

[B120] Reavie L. Buckley S. M. Loizou E. Takeishi S. Aranda-Orgilles B. Ndiaye-Lobry D. (2013). Regulation of c-Myc ubiquitination controls chronic myelogenous leukemia initiation and progression. Cancer Cell 23 (3), 362–375. 10.1016/j.ccr.2013.01.025 23518350 PMC3609428

[B121] Rossi D. (2019). FBXW7 is a biologically validated cancer driver gene for CLL. Blood 133 (8), 774–776. 10.1182/blood-2018-12-891507 30792223

[B122] Sailo B. L. Banik K. Girisa S. Bordoloi D. Fan L. Halim C. E. (2019). FBXW7 in cancer: what has been unraveled thus far? Cancers (Basel) 11 (2), 246. 10.3390/cancers11020246 30791487 PMC6406609

[B123] Sato H. Schoenfeld A. J. Siau E. Lu Y. C. Tai H. Suzawa K. (2020). MAPK pathway alterations correlate with poor survival and drive resistance to therapy in patients with lung cancers driven by ROS1 fusions. Clin. Cancer Res. 26 (12), 2932–2945. 10.1158/1078-0432.CCR-19-3321 32122926 PMC8034819

[B124] Senichkin V. V. Streletskaia A. Y. Gorbunova A. S. Zhivotovsky B. Kopeina G. S. (2020). Saga of Mcl-1: regulation from transcription to degradation. Cell Death Differ. 27 (2), 405–419. 10.1038/s41418-019-0486-3 31907390 PMC7206148

[B125] Shang W. Yan C. Liu R. Chen L. Cheng D. Hao L. (2021). Clinical significance of FBXW7 tumor suppressor gene mutations and expression in human colorectal cancer: a systemic review and meta-analysis. BMC Cancer 21 (1), 770. 10.1186/s12885-021-08535-8 34217244 PMC8254329

[B126] Sharma A. Singh K. Mazumder S. Hill B. T. Kalaycio M. Almasan A. (2013). BECN1 and BIM interactions with MCL-1 determine fludarabine resistance in leukemic B cells. Cell Death Dis. 4 (5), e628. 10.1038/cddis.2013.155 23681223 PMC3674362

[B127] Shen J. Z. Qiu Z. Wu Q. Zhang G. Harris R. Sun D. (2022b). A FBXO7/EYA2-SCFFBXW7 axis promotes AXL-mediated maintenance of mesenchymal and immune evasion phenotypes of cancer cells. Mol. Cell 82 (6), 1123–1139.e8. 10.1016/j.molcel.2022.01.022 35182481 PMC8934274

[B128] Shen W. Zhou Q. Peng C. Li J. Yuan Q. Zhu H. (2022a). FBXW7 and the hallmarks of cancer: underlying mechanisms and prospective strategies. Front. Oncol. 12, 880077. 10.3389/fonc.2022.880077 35515121 PMC9063462

[B129] Shimizu H. Takeishi S. Nakatsumi H. Nakayama K. I. (2019). Prevention of cancer dormancy by Fbxw7 ablation eradicates disseminated tumor cells. JCI Insight 4 (4), e125138. 10.1172/jci.insight.125138 30830867 PMC6478422

[B130] Siegel R. L. Miller K. D. Wagle N. S. Jemal A. (2023). Cancer statistics, 2023. CA A Cancer J. Clin. 73 (1), 17–48. 10.3322/caac.21763 36633525

[B131] Sigismund S. Avanzato D. Lanzetti L. (2018). Emerging functions of the EGFR in cancer. Mol. Oncol. 12 (1), 3–20. 10.1002/1878-0261.12155 29124875 PMC5748484

[B132] Song H. Liu D. Dong S. Zeng L. Wu Z. Zhao P. (2020b). Epitranscriptomics and epiproteomics in cancer drug resistance: therapeutic implications. Signal Transduct. Target Ther. 5 (1), 193. 10.1038/s41392-020-00300-w 32900991 PMC7479143

[B133] Song X. Shen L. Tong J. Kuang C. Zeng S. Schoen R. E. (2020a). Mcl-1 inhibition overcomes intrinsic and acquired regorafenib resistance in colorectal cancer. Theranostics 10 (18), 8098–8110. 10.7150/thno.45363 32724460 PMC7381732

[B134] Song Y. Lin M. Liu Y. Wang Z. W. Zhu X. (2019). Emerging role of F-box proteins in the regulation of epithelial-mesenchymal transition and stem cells in human cancers. Stem Cell Res. Ther. 10, 124. 10.1186/s13287-019-1222-0 30999935 PMC6472071

[B135] Song Y. Zhou X. Bai W. Ma X. (2015). FBW7 increases drug sensitivity to cisplatin in human nasopharyngeal carcinoma by downregulating the expression of multidrug resistance-associated protein. Tumour Biol. 36 (6), 4197–4202. 10.1007/s13277-015-3056-4 25586348

[B136] Strohmaier H. Spruck C. H. Kaiser P. Won K. A. Sangfelt O. Reed S. I. (2001). Human F-box protein hCdc4 targets cyclin E for proteolysis and is mutated in a breast cancer cell line. Nature 413 (6853), 316–322. 10.1038/35095076 11565034

[B137] Sui X. Chen R. Wang Z. Huang Z. Kong N. Zhang M. (2013). Autophagy and chemotherapy resistance: a promising therapeutic target for cancer treatment. Cell Death Dis. 4 (10), e838. 10.1038/cddis.2013.350 24113172 PMC3824660

[B138] Sulkshane P. Teni T. (2022). Myeloid cell leukemia-1: a formidable barrier to anticancer therapeutics and the quest of targeting it. Explor Target Antitumor Ther. 3 (3), 278–296. 10.37349/etat.2022.00083 36045907 PMC9400788

[B139] Sun Y. Nie W. Qiu B. Yang Q. Zhao H. (2022). FBXW7 affects autophagy through MCL1 in oral squamous cell carcinoma. Orig. Artic. Available at:https://onlinelibrary.wiley.com/doi/abs/10.1111/odi.14325 . 10.1111/odi.14325 38055341

[B140] Takeishi S. Matsumoto A. Onoyama I. Naka K. Hirao A. Nakayama K. I. (2013). Ablation of Fbxw7 eliminates leukemia-initiating cells by preventing quiescence. Cancer Cell. 23 (3), 34761 10.1016/j.ccr.2013.01.026 23518349

[B141] Takeishi S. Nakayama K. I. (2016). To wake up cancer stem cells, or to let them sleep, that is the question. Cancer Sci. 107 (7), 875–881. 10.1111/cas.12958 27116333 PMC4946711

[B142] Tamamouna V. Pavlou E. Neophytou C. M. Papageorgis P. Costeas P. (2022). Regulation of metastatic tumor dormancy and emerging opportunities for therapeutic intervention. Int. J. Mol. Sci. 23 (22), 13931. 10.3390/ijms232213931 36430404 PMC9698240

[B143] Tekcham D. S. Chen D. Liu Y. Ling T. Zhang Y. Chen H. (2020). F-box proteins and cancer: an update from functional and regulatory mechanism to therapeutic clinical prospects. Theranostics 10 (9), 4150–4167. 10.7150/thno.42735 32226545 PMC7086354

[B144] Tong J. Tan S. Nikolovska-Coleska Z. Yu J. Zou F. Zhang L. (2017b). FBW7-Dependent mcl-1 degradation mediates the anticancer effect of Hsp90 inhibitors. Mol. Cancer Ther. 16 (9), 1979–1988. 10.1158/1535-7163.MCT-17-0032 28619760 PMC5587378

[B145] Tong J. Tan S. Zou F. Yu J. Zhang L. (2017a). FBW7 mutations mediate resistance of colorectal cancer to targeted therapies by blocking Mcl-1 degradation. Oncogene 36 (6), 787–796. 10.1038/onc.2016.247 27399335 PMC5226932

[B146] Tong J. Wang P. Tan S. Chen D. Nikolovska-Coleska Z. Zou F. (2017c). Mcl-1 degradation is required for targeted therapeutics to eradicate colon cancer cells. Cancer Res. 77 (9), 2512–2521. 10.1158/0008-5472.CAN-16-3242 28202514 PMC5626525

[B147] Toribio M. L. González-García S. (2023). Notch partners in the long journey of T-ALL pathogenesis. Int. J. Mol. Sci. 24 (2), 1383. 10.3390/ijms24021383 36674902 PMC9866461

[B148] Tripathi V. Kaur E. Kharat S. S. Hussain M. Damodaran A. P. Kulshrestha S. (2019). Abrogation of FBW7α-dependent p53 degradation enhances p53's function as a tumor suppressor. J. Biol. Chem. 294 (36), 13224–13232. 10.1074/jbc.AC119.008483 31346036 PMC6737220

[B149] Wang H. Chen J. Zhang S. Zheng X. Xie S. Mao J. (2020). MiR-223 regulates autophagy associated with cisplatin resistance by targeting FBXW7 in human non-small cell lung cancer. Cancer Cell Int. 20, 258. 32577098 10.1186/s12935-020-01284-x PMC7304223

[B150] Wang H. Guo M. Wei H. Chen Y. (2021a). Targeting MCL-1 in cancer: current status and perspectives. J. Hematol. Oncol. 14, 67. 10.1186/s13045-021-01079-1 33883020 PMC8061042

[B151] Wang J. Li T. Wang B. (2021b). Exosomal transfer of miR-25-3p promotes the proliferation and temozolomide resistance of glioblastoma cells by targeting FBXW7. Int. J. Oncol. 59 (2), 64. 10.3892/ijo.2021.5244 34278448 PMC8295027

[B152] Wang J. Wang H. Peters M. Ding N. Ribback S. Utpatel K. (2019b). Loss of Fbxw7 synergizes with activated Akt signaling to promote c-Myc dependent cholangiocarcinogenesis. J. Hepatol. 71 (4), 742–752. 10.1016/j.jhep.2019.05.027 31195063 PMC6773530

[B153] Wang X. Zhang H. Chen X. (2019a). Drug resistance and combating drug resistance in cancer. Cancer Drug Resist 2 (2), 141–160. 10.20517/cdr.2019.10 34322663 PMC8315569

[B154] Wang X. Zhang J. Zhou L. Sun W. Zheng Z. G. Lu P. (2015). Fbxw7 regulates hepatocellular carcinoma migration and invasion via Notch1 signaling pathway. Int. J. Oncol. 47 (1), 231–243. 10.3892/ijo.2015.2981 25955618

[B155] Wang X. J. Yu J. Wong S. H. Cheng A. S. Chan F. K. Ng S. S. (2013). A novel crosstalk between two major protein degradation systems: regulation of proteasomal activity by autophagy. Autophagy 9 (10), 1500–1508. 10.4161/auto.25573 23934082

[B156] Wang Z. Fukushima H. Gao D. Inuzuka H. Wan L. Lau A. W. (2011). The two faces of FBW7 in cancer drug resistance. Bioessays 33 (11), 851–859. 10.1002/bies.201100101 22006825 PMC3364519

[B157] Wang Z. Liu P. Inuzuka H. Wei W. (2014). Roles of F-box proteins in cancer. Nat. Rev. Cancer 14 (4), 233–247. 10.1038/nrc3700 24658274 PMC4306233

[B158] Welcker M. Larimore E. A. Swanger J. Bengoechea-Alonso M. T. Grim J. E. Ericsson J. (2013). Fbw7 dimerization determines the specificity and robustness of substrate degradation. Genes Dev. 27 (23), 2531–2536. 10.1101/gad.229195.113 24298052 PMC3861666

[B159] Welcker M. Orian A. Grim J. E. Eisenman R. N. Clurman B. E. (2004). A nucleolar isoform of the Fbw7 ubiquitin ligase regulates c-Myc and cell size. Curr. Biol. 14 (20), 1852–1857. 10.1016/j.cub.2004.09.083 15498494

[B160] Wertz I. E. Kusam S. Lam C. Okamoto T. Sandoval W. Anderson D. J. (2011). Sensitivity to antitubulin chemotherapeutics is regulated by MCL1 and FBW7. Nature 471 (7336), 110–114. 10.1038/nature09779 21368834

[B161] Wilkinson L. Verhoog N. J. D. Louw A. (2018). Disease- and treatment-associated acquired glucocorticoid resistance. Endocr. Connect. 7 (12), R328–49. 10.1530/EC-18-0421 30352419 PMC6280593

[B162] Wood K. C. (2020). Overcoming MCL-1-driven adaptive resistance to targeted therapies. Nat. Commun. 11 (1), 531. 10.1038/s41467-020-14392-z 31988312 PMC6985132

[B163] Wu Z. H. Pfeffer L. M. (2016). MicroRNA regulation of F-box proteins and its role in cancer. Semin. Cancer Biol. 36, 80–87. 10.1016/j.semcancer.2015.09.016 26433073 PMC4761503

[B164] Xia W. Zhou J. Luo H. Liu Y. Peng C. Zheng W. (2017). MicroRNA-32 promotes cell proliferation, migration and suppresses apoptosis in breast cancer cells by targeting FBXW7. Cancer Cell Int. 17, 14. 10.1186/s12935-017-0383-0 28149200 PMC5267379

[B165] Xiao G. Li Y. Wang M. Li X. Qin S. Sun X. (2018a). FBXW7 suppresses epithelial-mesenchymal transition and chemo-resistance of non-small-cell lung cancer cells by targeting snai1 for ubiquitin-dependent degradation. Cell Prolif. 51 (5), e12473. 10.1111/cpr.12473 30094882 PMC6528938

[B166] Xiao Y. Yin C. Wang Y. Lv H. Wang W. Huang Y. (2018b). FBXW7 deletion contributes to lung tumor development and confers resistance to gefitinib therapy. Mol. Oncol. 12 (6), 883–895. 10.1002/1878-0261.12200 29633504 PMC5983212

[B167] Xie C. M. Sun Y. (2019). The MTORC1-mediated autophagy is regulated by the FBXW7-SHOC2-RPTOR axis. Autophagy 15 (8), 1470–1472. 10.1080/15548627.2019.1609864 31010381 PMC6613887

[B168] Xie C. M. Tan M. Lin X. T. Wu D. Jiang Y. Tan Y. (2019). The FBXW7-SHOC2-raptor Axis controls the cross-talks between the RAS-ERK and mTORC1 signaling pathways. Cell Rep. 26 (11), 3037–3050. 10.1016/j.celrep.2019.02.052 30865892 PMC6503676

[B169] Xing L. Xu L. Zhang Y. Che Y. Wang M. Shao Y. (2022). Recent insight on regulations of FBXW7 and its role in immunotherapy. Front. Oncol. 12, 925041. 10.3389/fonc.2022.925041 35814468 PMC9263569

[B170] Xu Z. Zhuang L. Wang X. Li Q. Sang Y. Xu J. (2020). FBXW7γ is a tumor-suppressive and prognosis-related FBXW7 transcript isoform in ovarian serous cystadenocarcinoma. Future Oncol. 16 (25), 1921–1930. 10.2217/fon-2020-0371 32915667

[B171] Yang H. Lu X. Liu Z. Chen L. Xu Y. Wang Y. (2015). FBXW7 suppresses epithelial-mesenchymal transition, stemness and metastatic potential of cholangiocarcinoma cells. Oncotarget 6 (8), 6310–6325. 10.18632/oncotarget.3355 25749036 PMC4467439

[B172] Yang Q. Zhao J. Chen D. Wang Y. (2021). E3 ubiquitin ligases: styles, structures and functions. Mol. Biomed. 2 (1), 23. 10.1186/s43556-021-00043-2 35006464 PMC8607428

[B173] Ye M. Zhang Y. Zhang X. Zhang J. Jing P. Cao L. (2017). Targeting FBW7 as a strategy to overcome resistance to targeted therapy in non-small cell lung cancer. Cancer Res. 77 (13):3527–39. 28522751 10.1158/0008-5472.CAN-16-3470

[B174] Yeh C. H. Bellon M. Nicot C. (2018). FBXW7: a critical tumor suppressor of human cancers. Mol. Cancer 17 (1), 115. 10.1186/s12943-018-0857-2 30086763 PMC6081812

[B175] Yeh C. H. Bellon M. Pancewicz-Wojtkiewicz J. Nicot C. (2016). Oncogenic mutations in the FBXW7 gene of adult T-cell leukemia patients. Proc. Natl. Acad. Sci. U. S. A. 113 (24), 6731–6736. 10.1073/pnas.1601537113 27247421 PMC4914202

[B176] Yeh C. H. Bellon M. Wang F. Zhang H. Fu L. Nicot C. (2020). Loss of FBXW7-mediated degradation of BRAF elicits resistance to BET inhibitors in adult T cell leukemia cells. Mol. Cancer 19 (1), 139. 10.1186/s12943-020-01254-x 32907612 PMC7487643

[B177] Yi X. Lou L. Wang J. Xiong J. Zhou S. (2021). Honokiol antagonizes doxorubicin resistance in human breast cancer via miR-188-5p/FBXW7/c-Myc pathway. Cancer Chemother. Pharmacol. 87 (5), 647–656. 10.1007/s00280-021-04238-w 33544209

[B178] Yokobori T. Mimori K. Iwatsuki M. Ishii H. Onoyama I. Fukagawa T. (2009). p53-Altered FBXW7 expression determines poor prognosis in gastric cancer cases. Cancer Res. 69 (9), 3788–3794. 10.1158/0008-5472.CAN-08-2846 19366810

[B179] Yokobori T. Yokoyama Y. Mogi A. Endoh H. Altan B. Kosaka T. (2014). FBXW7 mediates chemotherapeutic sensitivity and prognosis in NSCLCs. Mol. Cancer Res. 12 (1), 32–37. 10.1158/1541-7786.MCR-13-0341 24165483

[B180] Yu H. G. Wei W. Xia L. H. Han W. L. Zhao P. Wu S. J. (2013). FBW7 upregulation enhances cisplatin cytotoxicity in non-small cell lung cancer cells. Asian Pac J. Cancer Prev. 14 (11), 6321–6326. 10.7314/apjcp.2013.14.11.6321 24377525

[B181] Yu J. Zhang W. Gao F. Liu Y. X. Chen Z. Y. Cheng L. Y. (2014). FBW7 increases chemosensitivity in hepatocellular carcinoma cells through suppression of epithelial-mesenchymal transition. Hepatobiliary Pancreat. Dis. Int. 13 (2), 184–191. 10.1016/s1499-3872(14)60029-1 24686546

[B182] Zhang H. Chen F. He Y. Yi L. Ge C. Shi X. (2017). Sensitivity of non-small cell lung cancer to erlotinib is regulated by the Notch/miR-223/FBXW7 pathway. Biosci. Rep. 37 (3), BSR20160478. 10.1042/BSR20160478 28507201 PMC5479025

[B183] Zhang W. Koepp D. M. (2006). Fbw7 isoform interaction contributes to cyclin E proteolysis. Mol. Cancer Res. 4 (12), 935–943. 10.1158/1541-7786.MCR-06-0253 17189384

[B184] Zhao D. Zheng H. Q. Zhou Z. Chen C. (2010). The Fbw7 tumor suppressor targets KLF5 for ubiquitin-mediated degradation and suppresses breast cell proliferation. Cancer Res. 70 (11), 4728–4738. 10.1158/0008-5472.CAN-10-0040 20484041

[B185] Zhao J. Tang J. Men W. Ren K. (2012). FBXW7-mediated degradation of CCDC6 is impaired by ATM during DNA damage response in lung cancer cells. FEBS Lett. 586 (24), 4257–4263. 10.1016/j.febslet.2012.10.029 23108047

[B186] Zhong L. Zhang Y. Li M. Song Y. Liu D. Yang X. (2020). E3 ligase FBXW7 restricts M2-like tumor-associated macrophage polarization by targeting c-Myc. Aging (Albany NY) 12 (23), 24394–24423. 10.18632/aging.202293 33260160 PMC7762499

[B187] Zhong Q. Gao W. Du F. Wang X. (2005). Mule/ARF-BP1, a BH3-only E3 ubiquitin ligase, catalyzes the polyubiquitination of Mcl-1 and regulates apoptosis. Cell 121 (7), 1085–1095. 10.1016/j.cell.2005.06.009 15989957

[B188] Zhou P. Li B. Liu F. Zhang M. Wang Q. Liu Y. (2017). The epithelial to mesenchymal transition (EMT) and cancer stem cells: implication for treatment resistance in pancreatic cancer. Mol. Cancer 16 (1), 52. 10.1186/s12943-017-0624-9 28245823 PMC5331747

[B189] Zhou X. Jin W. Jia H. Yan J. Zhang G. (2015b). MiR-223 promotes the cisplatin resistance of human gastric cancer cells via regulating cell cycle by targeting FBXW7. J. Exp. Clin. Cancer Res. 34 (1), 28. 10.1186/s13046-015-0145-6 25888377 PMC4387683

[B190] Zhou Z. He C. Wang J. (2015a). Regulation mechanism of Fbxw7-related signaling pathways (Review). Oncol. Rep. 34 (5), 2215–2224. 10.3892/or.2015.4227 26324296

[B191] Zhu T. Liu B. Wu D. Xu G. Fan Y. (2021). Autophagy regulates VDAC3 ubiquitination by FBXW7 to promote erastin-induced ferroptosis in acute lymphoblastic leukemia. Front. Cell Dev. Biol. 9, 740884. 10.3389/fcell.2021.740884 34869326 PMC8634639

[B192] Zou Q. Liu M. Liu K. Zhang Y. North B. J. Wang B. (2023). E3 ubiquitin ligases in cancer stem cells: key regulators of cancer hallmarks and novel therapeutic opportunities. Cell Oncol. (Dordr) 46 (3), 545–570. 10.1007/s13402-023-00777-x 36745329 PMC10910623

